# Maculopathies: A Systematic Literature Review on Pathophysiology, Public Health, and Treatment

**DOI:** 10.7759/cureus.74911

**Published:** 2024-12-01

**Authors:** Maria E Pantelidou, David Sunnucks, Elias P Pantelidis

**Affiliations:** 1 Ophthalmology, James Paget University Hospital, Great Yarmouth, GBR; 2 Barts and the London School of Medicine and Dentistry, Queen Mary University of London, London, GBR; 3 Ophthalmology, Ramsay Health Care, Mount Stuart Hospital, Torquay, GBR

**Keywords:** adult-onset foveo-macular vitelliform dystrophy, healthcare sustainability, maculopathy, physical and psychological well-being, stargardt disease, stem-cell transplant, vision impairment

## Abstract

Macular degeneration (MD) is a pathological condition affecting the macula, an area located near the center of the retina. This disease affects individuals of all ages, both children and adults, causing severe visual impairment. Age-related macular degeneration (AMD) is the leading cause of visual loss in the older population while Stargardt disease (SD) is the most common hereditary maculopathy with an autosomal dominant pattern of inheritance. Current management involves anti-vascular endothelial growth factor intravitreal injections, visual aids, and other conservative prevention mechanisms that can only delay the inevitable progress of the disease. Macular dystrophies have an impact on both individuals and societies with psychological and financial implications, respectively. It is evident that vision impairment has a significant impact on patients' physical and mental well-being, and therefore it is important to improve current treatment modalities, develop stem cell therapies, and further novel treatments in order to provide a better prognosis and overall quality of life.

## Introduction and background

This review aims to present the current knowledge on the various types of maculopathies and discuss current and future treatment modalities of this ophthalmic pathology. This review will touch mainly on age-related macular degeneration (AMD), diabetic maculopathy, and a variety of congenital macular dystrophies. The pathophysiology of these conditions will be presented in order to show the variety of the pathology of these diseases. Standard and future treatment modalities will also be discussed, and a sustainability perspective of the vision loss related to maculopathies will be attempted. The importance of maculopathies is related to their prevalence and their significant impact on vision. It is estimated that in 30 years, globally, there will be 17.8 million patients with AMD who will suffer from irreversible vision loss [1,2]. Management comprises anti-VEGF injections and laser treatments to control the ongoing pathology. Novel treatments involve stem cell research and are currently in the early clinical trial stage [3]. The prevalence of various types of maculopathies is different. It is estimated that one in 10K of the individuals presenting with an ophthalmic condition, in the first two decades of their life, are diagnosed with a maculopathy [4,5]. The term maculopathy involves the terms macular dystrophy and MD. The first usually describes congenital maculopathies in the early years, and the second describes maculopathies that are age-related and present in the form of degeneration later in life [6].

Maculopathy is an ophthalmic condition characterized by progressive deterioration that results in a central lesion in the macular area of the eye that causes abrupt, non-reversible changes, while the peripheral vision remains intact [7].

In adulthood, MD predominantly affects individuals above the age of 65, with a prevalence in the US being 1.47%. The pathogenesis is multifactorial and intricate, due to the underlying cellular complexity of the retina. Genetic mutations in the bestrophin-1 (BEST1) gene are more commonly associated with hereditary ophthalmic conditions, which have collectively been referred to as bestrophinopathies. Lately, further knowledge regarding the genetic background and the complexity of dry AMD has evolved. However, the treatment continues to be elusive, despite some early biochemical suppression of inflammation and stem cell-based treatment modality exploration [8].

Diabetic maculopathy is the most prevalent cause of visual impairment in those with diabetic retinopathy. Systemic risk factors include higher systolic blood pressure, higher hemoglobin A1C, and higher lipid levels. In addition, diabetic maculopathy is the leading cause of blindness among adults in the working-age group. The severity of diabetic retinopathy is the only recognized ocular risk factor for diabetic maculopathy [9]. Management involving anti-VEGF agents (intravitreal anti-vascular endothelial growth factor injections), focal modified grid laser (local cauterization of ocular blood vessels), and rarely vitrectomy (removal of the vitreous humor of the eye) has been suggested. However, further research to explore the genetic basis of diabetic macular edema (DME) resistance in diabetic retinopathy patients and clinical research for the development of new treatment modalities are expected to provide an enhancement of a rational basis for additional effective therapies [10-13].

Macular dystrophies are a group of congenital macular diseases that cause macular atrophic pathology and significant visual loss from the early years. Although the primary findings are localized to the macula, electrophysiological and histological evidence suggests a broader contribution of the retina to the pathology [14]. Over the last two decades, there have been significant innovations in the comprehension of the genetic basis and the related pathophysiological mechanisms for each subdivision of macular dystrophies. Despite these advancements, effective treatments remain unavailable for these congenital diseases. However, stem cell-based research has demonstrated a promising safety profile and offers hope for future therapies that could slow or prevent progressive visual loss, or potentially restore some central vision [15,16].

## Review

Methods

**Figure 1:**
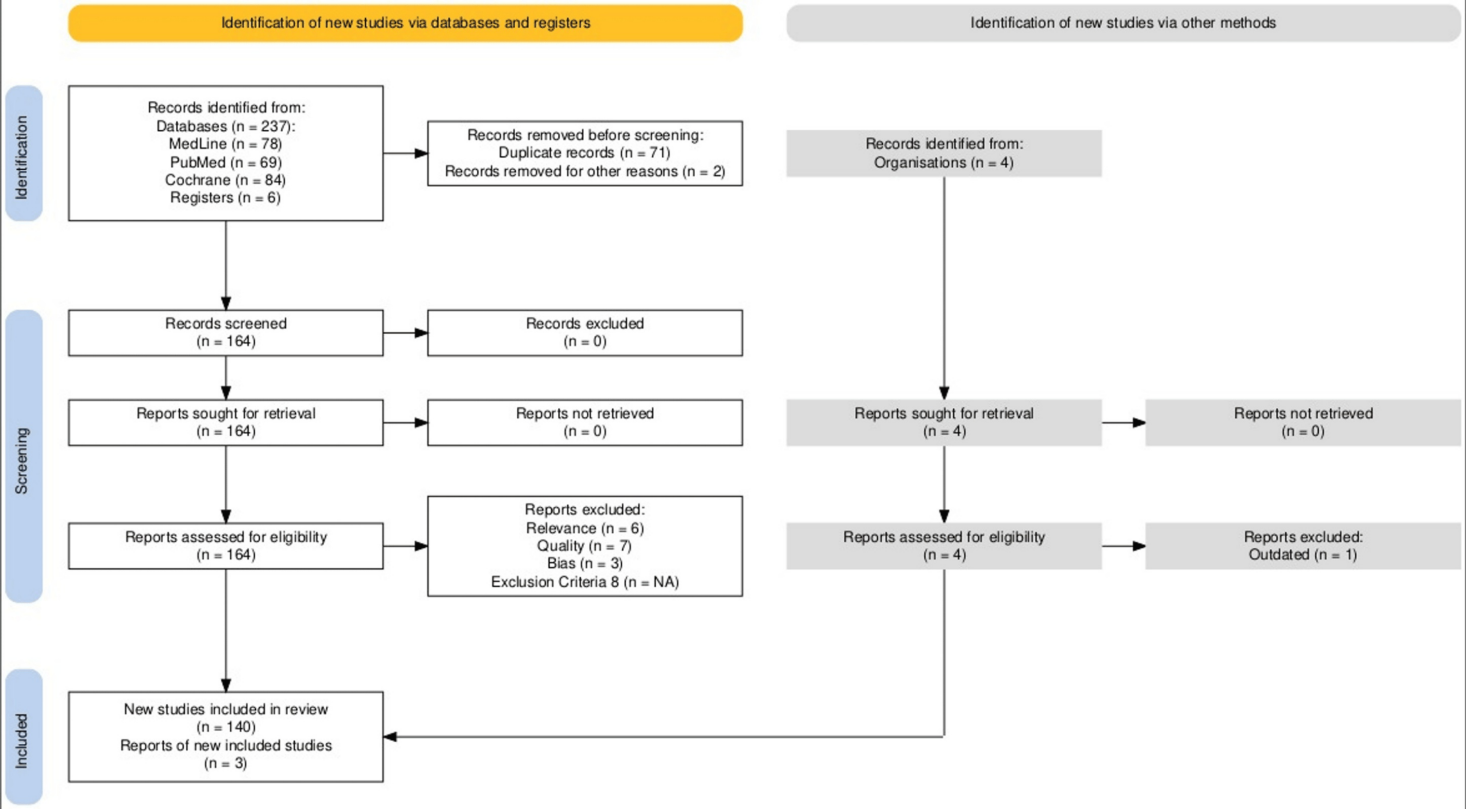
Preferred Reporting Items for Systematic Reviews and Meta-analyses (PRISMA) 2020 flow chart for the literature selection process of this review This chart follows the latest standards of the PRISMA flow chart and has been created according to PRISMA's latest free online software.

This systematic review was conducted following principles of the Preferred Reporting Items for Systematic Reviews and Meta-analyses (PRISMA) guidelines (Figure 1).

Literature Search Strategy

A literature search was performed from January to May 2024 using Medline, PubMed, Cochrane Library, and Web of Science and searching relevant articles from the past until the most recent publications. These four databases were chosen as they cover most of the literature in the fields of maculopathy and medicine. Two authors (MP and EP) performed the search using the keywords “maculopathy”, “anti-VGF injection”, “macular degeneration”, “Stargardt disease”, “vision loss”, and “vision sustainability”. The search was limited to English language documents. The keywords were matched with MeSH (Medical Subject Headings) terms and subheadings when relevant. Truncations and wildcards were utilized to capture all forms of a root word.

Inclusion and Exclusion Criteria

The process of determining study eligibility was undertaken by two independent review authors, using a consensus procedure for any disagreements, at both title/abstract and full-text screening stages. Two authors (MP and EP) screened citations and selected studies. In the first level of screening, titles and abstracts were reviewed, and potentially relevant articles were retrieved and assessed for inclusion. Any disagreement or ambiguity was resolved by consensus. For each eligible study, data elements were extracted from full-text versions by one author, and a second author verified the extractions with the original sources. The final selection of full-text articles was based on the following publication type inclusion/exclusion (Table 1).

**Table 1:** Inclusion and exclusion criteria

Section	Inclusion	Exclusion
Publication type	Peer-reviewed journal articles	No peer-reviewed journal articles
	Studies published in English	Survey studies
	Full-text articles	Review studies
	Validated textbooks and online eye encyclopedias	

Risk of Bias and Quality Assessment

Most of the inclusions were peer-reviewed journal articles, which upheld the quality of the systematic review and reduced the risk of publication bias. The quality assessment was performed at the study level. Two independent reviewers assessed all articles that met the inclusion criteria. In addition, a quality assessment was performed using a customized quality questionnaire with the following questions: 1. Were the study objectives clearly stated? 2. Was the study design clearly described? 3. Were key findings supported by the results? 4. Were the limitations of the study clearly described? 5. Were key findings supported by other literature? 6. Were conclusions drawn from the study clearly stated?

This approach was based on standardized methods and reviews, and it allowed for clinical information extraction that is relevant to the topic of interest.

Results

Age-Related Macular Degeneration

AMD is a disease of the macular area, most often clinically apparent in individuals above the age of 50 years. It is characterized by early- and late-stage pathology, which displays differences in the levels of retinal pigmentation. More specifically, early AMD characteristics include discrete yellow spots at the macula (drusen), hyperpigmentation of the retinal pigment epithelium (RPE) with evident demarcated areas, whereas late AMD shows geographic atrophy of the photoreceptors and the RPE with visible underlying choroidal vessels, evident choroidal neovascularization (CNV), and RPE detachments with or without neurosensory detachment. Scar tissue, exudates, and hemorrhage may also be relevant. AMD can be atrophic, following a progressive pattern with visible drusen and geographic atrophy of the RPE and exudative, eventually resulting in a fibroglial subretinal scar tissue formation. These two patterns carry the terms dry or non-exudative and wet or neurovascular, respectively [17-19].

Prevalence of AMD: AMD is the most common cause of irreversible visual loss in the Western world in people over the age of 50 years. The prevalence of severe visual loss increases proportionally with age. In particular, 10% of individuals in the US between the ages of 65 and 75 have lost some portion of their vision secondary to AMD. For those over 75 years, 30% are affected to some degree, while end-stage AMD was noted in 1.7% of all individuals above the age of 50 and 18% of those over the age of 85 years [20].

Hereditary Maculopathies

The inherited MDs comprise a heterogeneous group of disorders characterized by macular atrophy, central visual impairment, and RPE atrophy [21]. The main characteristics include significant bilateral visual loss explained by symmetrical macular anomalies in both eyes and clinically observed by fundoscopy. The age onset is variable but is usually summarized within the first two decades of life. Heterogeneity is also found in the genetic inheritance of the different types as MDs present in multiple modes of inheritance (autosomal dominant, autosomal recessive, x-linked recessive, and mitochondrial inheritance). However, the diagnosis of inherited retinal degeneration (IRD) is challenging due to the heterogeneity of the genotype and phenotype that it presents with [14]. In a 2021 cohort study in Taiwan, IRD cases were identified to be a genetic-related disease in 57.1% of the recruited population, with ATP-binding-cassette-subfamily-a-member-4 (ABCA4) variants being the most common at 15.2% and cytochrome-P450-family-4-subfamily-V-member-2 (CYP4V2) variants the most abundant cause of single phenotype (3.8% Bietti’s crystalline dystrophy). Stargardt disease (STGD) and fundus flavimaculatus (FF) are considered different variants of the same condition regardless of the different times and different prognoses they present with [14].

Stargardt maculopathy (juvenile MD): Stargardt MD (STGD) accounts for 7% of all retinal degenerations and is the most common inherited maculopathy with a prevalence of one in 10,000 and an autosomal recessive mode of genetic inheritance. The gene locus is ABCA4 in 1p21-22. As with all autosomal cases, males and females are equally affected and no race predilection has been detected [14]. As with most genetically related MDs, STGD classically presents in the first to the second decade of life with bilateral gradual impairment of central vision, which can be disproportionate to the underlying macular changes [22-24].

This is a gradually evolving condition where signs develop according to the different stages of the disease (Table 2) [25].

**Table 2:** Fishman classification of Stargardt disease The four stages of the classification of Stargardt disease by Fishman [25]. Abbreviations: DD: disc diameter, EOG: electro-oculogram, ERG: electroretinogram

Stage	Description
Stage 1	Pigmentary changes in the macula (ranging from faint/irregular pigment mottling to a beaten-bronze appearance to atrophy). Pisciform ring of flecks within 1DD on all sides of the fovea. Normal ERG and EOG.
Stage 2	Pisciform flecks present beyond 1DD from the fovea, often extending beyond the arcades and nasal to the optic disc. ERG and EOG normal, but cone/rod response may be subnormal. Prolonged period for dark adaptation.
Stage 3	Diffusely resorbed flecks and choriocapillaris atrophy in the retina. EOG: subnormal ratios. ERG: subnormal cone or cone and rod amplitudes. Central field defects and midperipheral/peripheral field impairments.
Stage 4	Diffusely resorbed flecks and extensive choriocapillaris/RPE atrophy throughout entire fundus. ERG: reduced cone and rod amplitudes. Peripheral field show moderate to extensive restriction.

**Figure 2:**
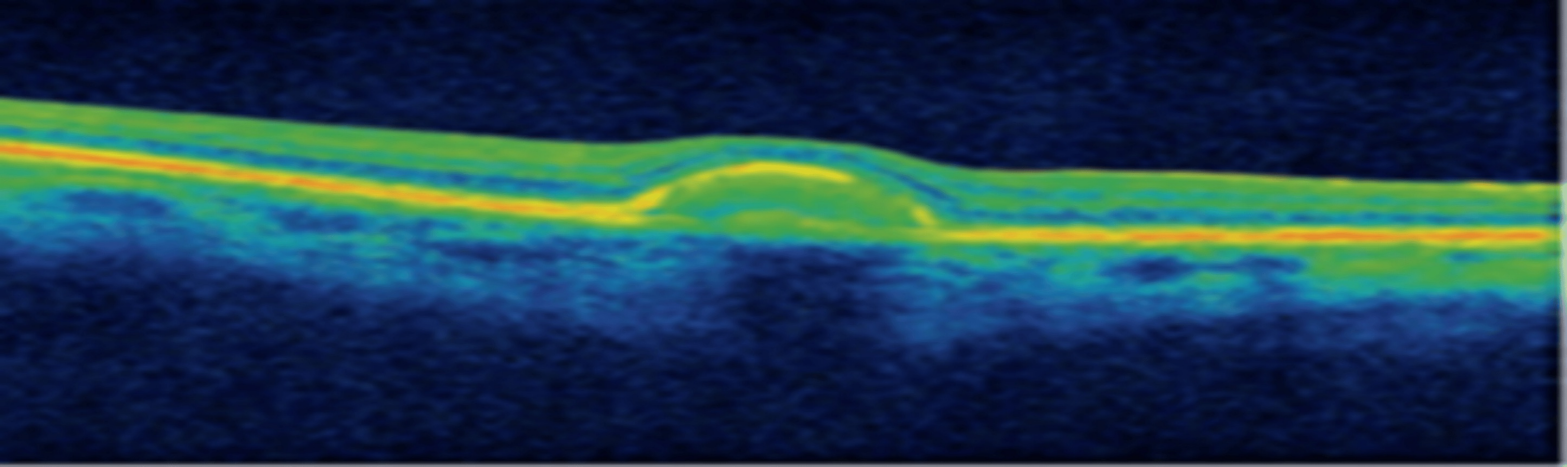
Ocular coherence tomography (OCT) showing abnormal material within and above the retinal pigment epithelium (RPE) Source: [27] (Permission was obtained from the original publisher)

**Figure 3:**
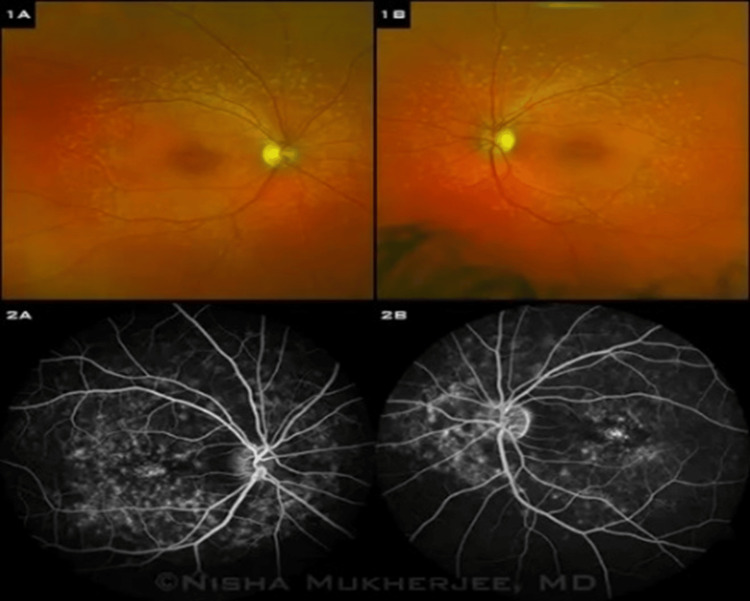
Stardgardt disease: A. fundus photograph, B. fluorescein angiography Source: [28] (Permission was obtained from the original publisher)

Pathophysiologically, it is characterized by deposits of lipofuscin within the RPE (Figure 2). In the early stages, the fovea might be clear or show non-specific mottling before the appearance of white-yellow flecks in the surrounding area (Figure 3A). Fluorescein angiography (Figure 3B) characteristically reveals a dark choroid, appearing in 80% of cases, highlighting the visualization of the circulation from the choroidal capillaries, which are secondary to excess lipofuscin deposits within the RPE. The mutations in ABCA4 result in the accumulation of all-trans-retinal in the photoreceptors and the RPE. Mutations in ABCA4, elongation-of-very-long-chain fatty acids-like 4 (ELOVL4), and peripherin-2 (PRPH2) are the etiology of 95% of STGD [26-28].

Color vision shows an absence or abnormal photopigments (deutan-tritan defects) as an early sign and therefore should be considered. Photopic electroretinogram (ERG, a diagnostic test used to measure the electrical activity of the retina in response to a light stimulus) can be either normal or abnormal, scotopic is normal, and an electrooculogram (EOG) indicates subnormal signs to predominantly advanced stages of the maculopathy.

The prognosis is poor since there is no effective treatment for the RPE changes, and once visual acuity (VA) reaches 6/12, it tends to decrease and stabilize at 6/60. However, the phenotypic variability, with over 490 disease-associated variants discovered so far, is imprinted on the highly variably visual function of everyone. A study that aimed to explore the diagnostic value of electroretinography showed accurately that the probability of maintaining a VA of 20/40 or higher, at least unilaterally, was 52% by the age of 19, 32% by the age of 29, and decreased to 22% by the age of 39 years [29].

Counseling is important for patients. Giving up smoking and avoiding sun exposure are advised since both of them have been found to result in the formation of trans-retinal photoreceptors and contribute to further lipofuscin accumulation. High doses of vitamin A intake may also lead to deterioration of the condition (National Eye Institute, 2021) [30,31].

**Figure 4:**
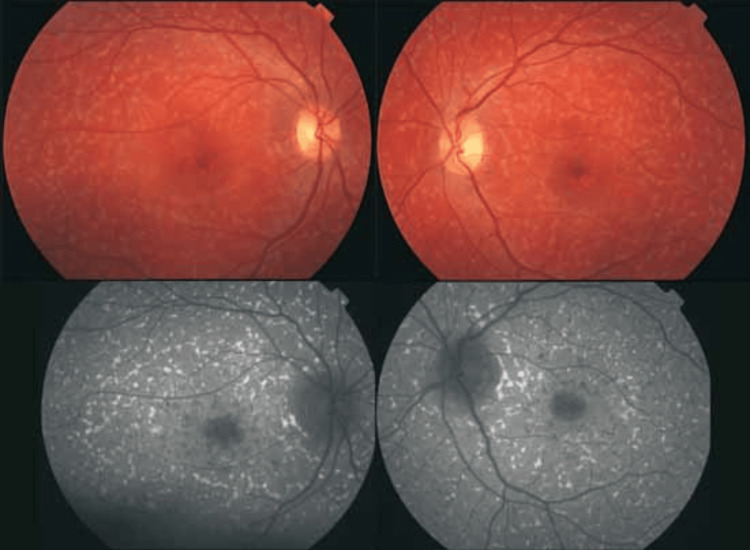
Central hypo-autofluorescent region with extensive hyper-autofluorescent flecks all over the fundus (fundus flavimaculatus) Source: [34] (Permission was obtained from the original publisher)

Fundus flavimaculatus: FF shares obvious similarities with STGD and is accepted that both conditions are genetically linked. Patients with FF often have a later presentation onset and a slower visual deterioration, reporting this condition as milder [32,33]. As in adult life, in the absence of macular involvement, the condition may be asymptomatic and discovered by chance. Bilateral, yellow-white freckles are also present (Figure 4), scattered at the posterior pole and the mid-periphery of RPE, and may have a variety of shapes (oval, linear, semilunar, and disciform) [34].

The prognosis is relatively good, and patients may remain asymptomatic for many years unless one of the flecks involves the foveal center (foveola) or geographic atrophy develops [25]. The Hereditary Ocular Disease Database of the University of Arizona Health reported a more promising prognosis in VA in later-onset disease, in relevant research involving patients with MD. In particular, 23% presented with 20/40 VA, 20% with 20/50-20/100 VA, 55% with 20/200-20/400 VA, and a small number had a vision of less than 20/400 VA [35]. Some studies review STGD and FF under the same scope, due to the limited diagnostic distinctions of the wide range of non-specific clinical manifestations [34,36,37].

**Figure 5:**
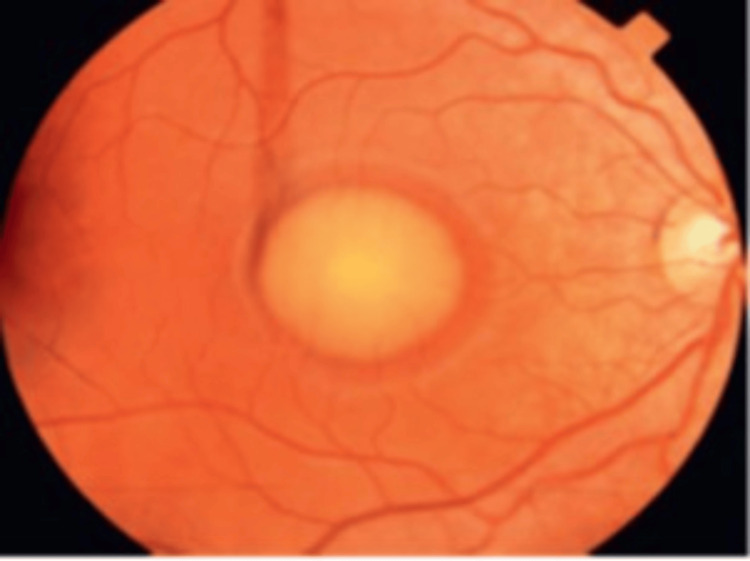
Vitelliform Best disease Source: [39] (Permission was obtained from the original publisher)

**Figure 6:**
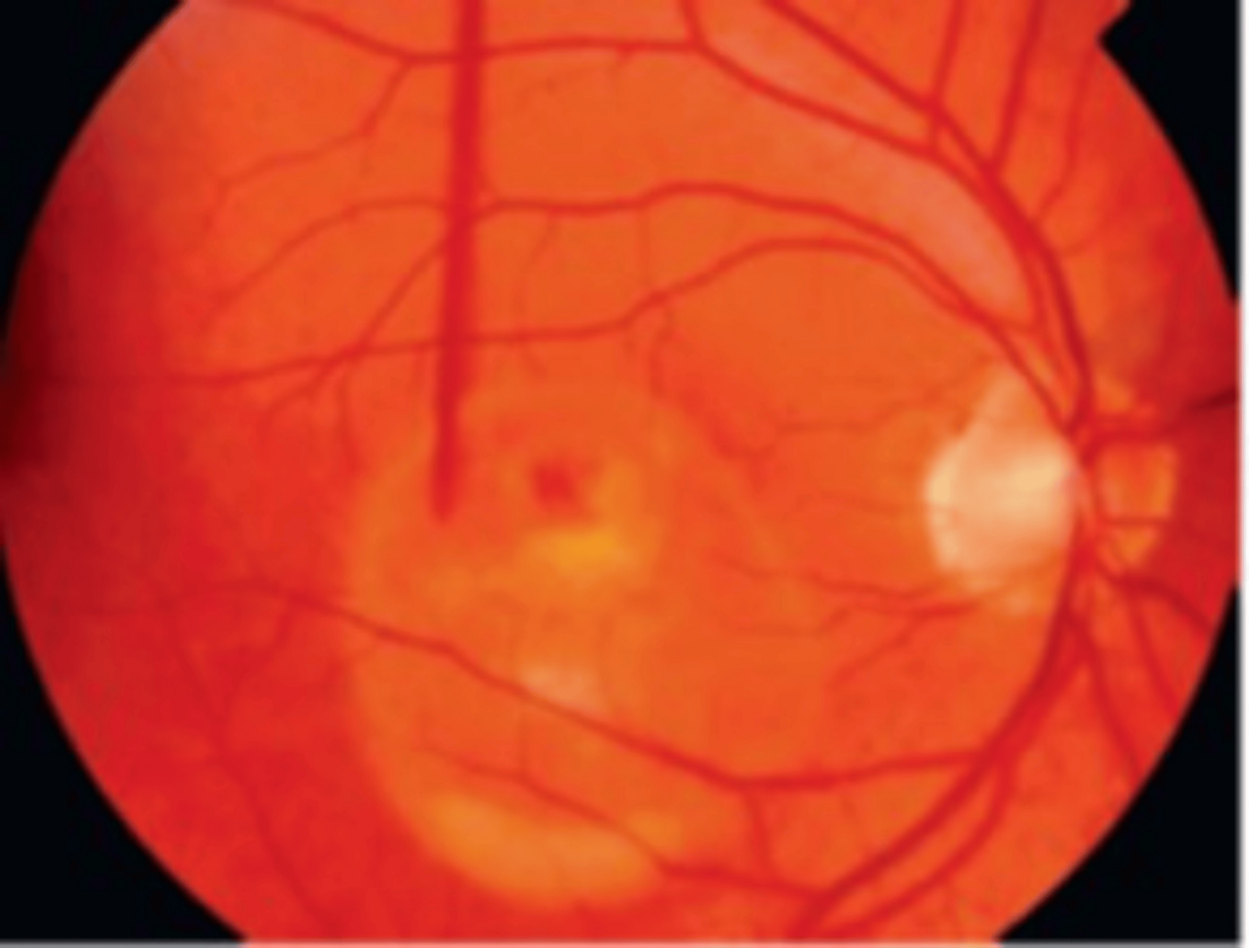
Pseudo-hypopyon Best disease Source: [39] (Permission was obtained from the original publisher)

**Figure 7:**
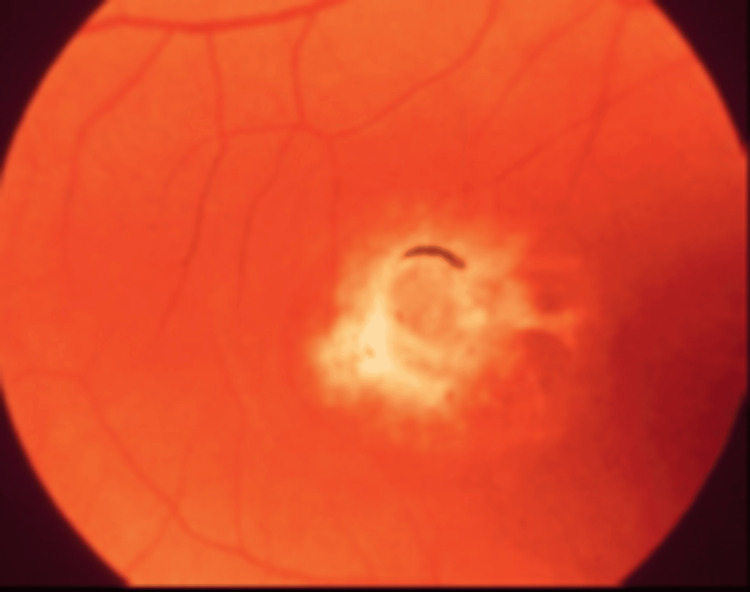
Viteliruptive Best disease Source: [39] (Permission was obtained from the original publisher)

**Figure 8:**
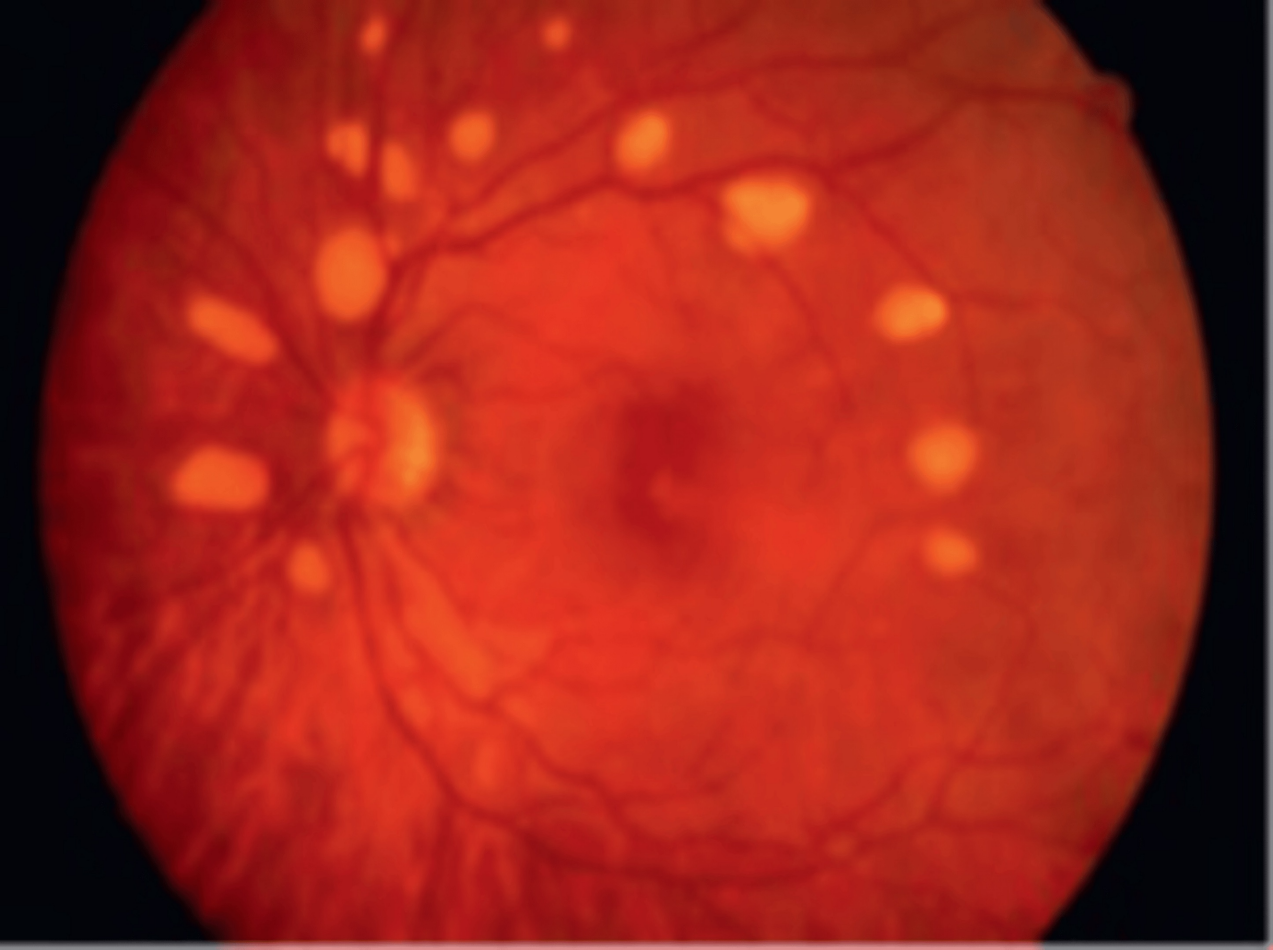
Multifocal vitelliform lesions without Best disease Source: [39] (Permission was obtained from the original publisher)

Vitelliform dystrophy (juvenile Best disease): Juvenile vitelliform macular dystrophy (VMD) is a rare autosomal dominant condition evolving gradually through five stages. VMD occurs primarily in European Caucasians with only one copy of the vitelliform-macular-dystrophy-2 (VMD2) gene located in chromosome 11q13, sufficient for the development of VMD [38]. It is characterized clinically by the classical feature of a round or oval yellow subretinal macular deposit, which mimics an egg yolk and is gradually reabsorbed over time. There is clinical appearance variation, as shown in Figures 5-8.

Histopathological research on donated eyes has shown accumulation of lipofuscin throughout the RPE evident in an EOG examination [39,40]. Lipofuscin is a fluorescent pigment formed in several compartmental tissues but abundantly studied in the eyes. Essentially, the accumulation of lipofuscin in the RPE is a characteristic mark of aging in the eye and is evaluated by a pigment-sensitive test (EOG), which measures the standing potential between the cornea (electrically positive) and the posterior eye (electrically negative) [26]. In a study conducted in Olmsted County, Minnesota, the prevalence was noted to be one in 16,5000 to one in 21,000 with males and females equally affected. Prognosis is reasonably good until the fifth decade, after which VA declines and some patients become blind because of macular scarring [41].

Adult foveomacular vitelliform dystrophy: Adult foveomacular vitelliform dystrophy (AFVD) is an autosomal dominant condition with the gene locus on 6p21-22, at its peak usually between 30 and 50 years of age. The classical description of the presentation involves bilateral, symmetrical, slightly elevated, yellow sub-foveal lesions about one-third disc diameter in size. Although the pathophysiology is still unknown, it has been speculated that abnormal lipofuscin accumulation caused by the increased metabolism and phagocytosis on the RPE in conjunction with environmental factors may have an effect. Currently, known genetic causes include mutations in the PRPH2, BEST1, interphotoreceptor-matrix-proteoglycan IMPG1, and IMPG2 genes. Diagnostic tests ERG and EOG appear normal, but color vision has been reported to be impaired (tritan defect) [42].

The prevalence of AFVD is unknown. The prognosis is good, as the disease causes gradual visual loss with late-onset and the vitelliform lesion typically resolves later in life [43].

Multifocal Best disease: Multifocal Best disease is a very unusual disease characterized by multiple demarcated yellow lesions located in the posterior pole of the RPE. Many cases present without a family history. The prevalence is not known [44].

Doyne honeycomb choroiditis (familial Drusen or Malattia Levantinese): Familial drusen (FD) is an early manifestation of AMD. It is a condition with an autosomal dominant inheritance pattern with full penetrance but variable expressively, and the gene locus EGF-containing-fibulin-extracellular-matrix-protein-1 (EFEMP1) is on 2p16. Symptoms are experienced between the fourth and fifth decades of life and include initially innocuous hard drusen confined to the macula that can develop into geographic atrophy and CNV at a later stage [45]. An animal study has suggested that the EFEMP1 mutation is both pathological and responsible for the deposit developments between Bruch’s membrane and RPE in mice and that the basal deposits were responsible for modifications in the RPE cell ultrastructure and were linked to higher levels of C3 complement activation [46]. A 2005 paper investigating 600 eyes showed the prevalence of drusen in different age groups to be increasing as the age increased; 0% in the age range 20-24 years; 35.9% aged 25-29; 23.7% aged 30-34; 35.9% aged 35-39; 47.2% aged 40-44; and 48.6% aged 45-49 years [47].

In agreement with the above, a 2012 UK study aimed to describe the prevalence of pigmentary changes using the Wisconsin-related maculopathy grading system in 500 middle-aged individuals. Drusen were identified within the central macular grid in 91.48% of all eyes examined and drusen sized less than 31.5 μm were present in 89% of eyes, with 45.9% of eyes having larger-sized drusen [48]. Although these studies provide important data with regard to drusen prevalence, hard drusen are considered a normal presentation in most adults with no reported family history or syndromes; however, soft drusen appear to be more related to a genetic component, AMD in particular [49].

Malattia Levantinese shares a phenotypic overlap with FD and is characterized by small, innumerable basal laminar drusen with a spoke-like or radial distribution centered at the fovea and parapapillary area of the eye. Most patients are asymptomatic until the development of geographic atrophy and CNV [50].

**Figure 9:**
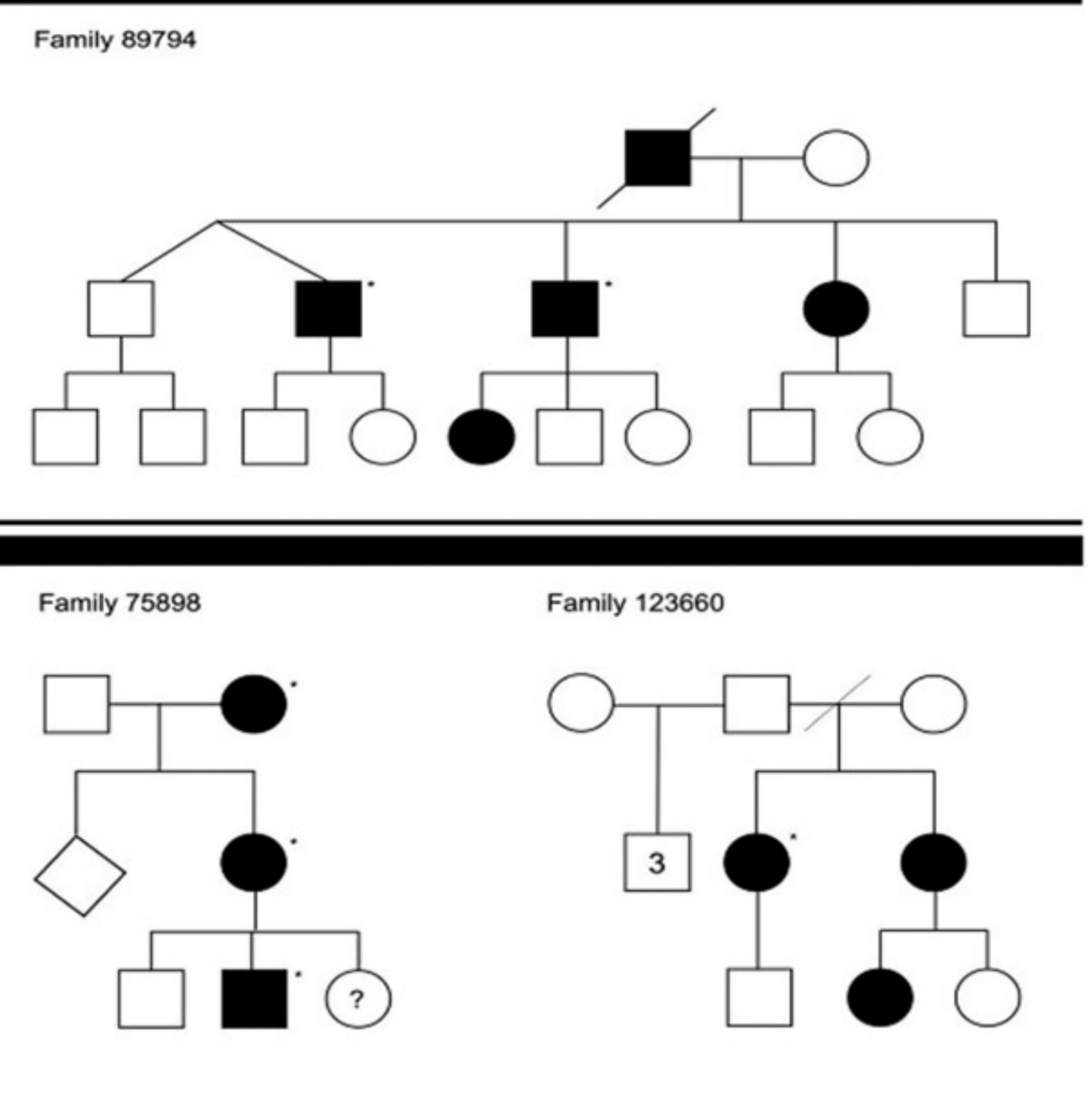
Pedigrees from three families segregating North Carolina macular dystrophy (NCMD). The study participants are highlighted with asterisks Source: [60] (Permission was obtained from the original publisher)

North Carolina macular dystrophy: North Carolina macular dystrophy (NCMD) is a very rare but severe macular disease. It is an autosomal dominant condition with complete penetrance but highly variable expressivity (Figure 9) with the gene macular-dystrophy-retinal-1 (MCDR1) on the 6q chromosome. Manes et al. described a novel duplication of PR/SET-domain-13 (PRMD13) causing NCMD, supporting even further the genetic linkage of duplications on the entire sequence of cyclin-c (CCNC) and PRDM13 genes with this degeneration. Although some researchers consider NCMD non-progressive, others support it may progress slowly until the age of 12, if further complications including CNV, occur [51-54]. The phenotype ranges from subtle drusen and macular sports to bilateral end-stage coloboma-like atrophic macular lesions with variable impairment in VA [51,55-59]. NCMD was first described in the 1970s in a large pedigree of more than 500 individuals spanning seven generations from two brothers in Ireland. Since the condition was first described, families have been reported in North America, Asia, Europe, and elsewhere globally [60,61].

Butterfly macular dystrophy: Butterfly macular dystrophy (BMD) is a rare and relatively innocuous condition. It is one of the five subtypes of MD patterns and affects the RPE with yellow pigments arranged in a triradiate manner at the fovea. Pigmentary stippling in the periphery may be present. ERG and EOG are usually normal, and the FA shows the corresponding hypofluorescence, which can help distinguish the different types [48-50]. According to a 1991 observational study, BMD has a rare incidence and prevalence of approximately four million people in a research study that spanned 18 years in Northern France. In 1,660 patients with diagnosed inherited retinal dystrophies, only three were identified with BMD, which gives a prevalence of 75 x 10^8^ among all the study subjects [60,62].

Dominant cystoid macular edema: Dominant cystoid macular edema (DCME) is an extremely rare but serious condition with an autosomal dominant genetic inheritance and the gene locus on 7q. Presentation occurs in the first two decades of life with cystic spaces in the macula leading to gradual impairment of central vision. Prognosis is poor, because of the eventual development of geographic atrophy [60,63]. A correlation with retinitis pigmentosa-1-like-1 (RPILI) mutation was also reported in the third decade of life. All various presentations of DCME involve mainly the macular area while the rest of the retina is spared [64]. Liew et al. in their 2012 research project aimed to report the prevalence of treatable ophthalmic complications, including cystoid macular edema, which was noted in 58.6% and 50.9% of cases to be bilateral. DCME was associated with younger age but not with cataracts, pseudophakia, and gender [65].

Diabetic Maculopathy

Diabetic maculopathy is the leading cause of visual deterioration as a complication of diabetic retinopathy in diabetic individuals, more often non-dependent on insulin. The literature demonstrates three main categories, exudative (focal), edematous (cystoid or diffuse) and ischemic. Although the absolute presentation can be different among the categories, all types have the same etiology and mode of treatment approach, and as macular edema is the dominant clinical sign, the condition is frequently referred to as DME [66].

Prevalence of DME: The most common cause of vision loss from diabetes mellitus is DME [67]. In the US, the epidemiological weighting of DME is 3.8% or 746 million individuals over the age of 40 years, ranking higher for those with type 1 diabetes mellitus (14%) than those with type 2 diabetes mellitus (6%) in worldwide pooled data [68]. Incidence rates of DME are not broadly explored. The Wisconsin Epidemiologic Study of Diabetic Retinopathy found that approximately 20% of patients with type 1 diabetes mellitus and 15-25% of patients with type 2 diabetes mellitus developed DME in a timeline of 10 years and 29% of type 1 diabetes mellitus patients developed DME over 25 years. Future data showed an improvement considering the relation between vision loss and diabetes mellitus which was attributed to advancements in healthcare [69].

The Los Angeles Eye Study compared Mexican Americans (MA) to non-Hispanic White (NH) individuals and found a much higher progression of diabetic retinopathy and DME in the MA population, showing the involvement of ethnicity in DME percentages [70]. An earlier study of US MA found a four-year cumulative incidence of diabetic retinopathy of 22.5% and a progression rate of 24.1% [11]. The paradox is that, although the rates of body mass index (BMI) presentation may be declining in individuals with diabetes mellitus, the numbers of DME cases still increase linearly due to the newly diagnosed patients with diabetes mellitus, leading to an overall increase of DME incidence [71].

Pathophysiology of DME: DME is the physiological continuation of diabetic retinopathy. As a disease, retinopathy exhibits features of microvascular occlusion and leakage. The vascular modifications result in decreased perfusion, hypoxia, and eventually retinal ischemia. The breakdown of the inner barrier of the retina leads to leakage of plasma concentration into the retina. As the accumulation of extracellular fluid occurs within the retina and is reabsorbed into the retinal capillaries, the focal compilation of large molecules is unable to be removed, giving the classic picture of plaque-like hard exudates. Those exudates can either be absorbed spontaneously for months or years or remain in situ and accumulate if chronic leakage takes place. Hard exudates are mainly composed of lipids and proteins and can settle in positions in the macula, with the foveal region being the most important regarding visual loss [10,72].

Drug-Induced Maculopathy

Antimalarials: Chloroquine and hydroxychloroquine are quinolone derivatives used in the prophylaxis and treatment of malaria, as well as in other systematic diseases, such as rheumatoid arthritis, systemic lupus erythematosus, and sarcoidosis. Antimalarials are melanotropin drugs that become concentrated in structures containing melanotropin cells such as the RPE and the choroid. The two main ocular effects of the antimalarials are retinotoxicity and corneal deposits. Severe complications include vortex keratopathy, an extremely uncommon retinal change [73].

Chloroquine retinotoxicity is related to the total cumulative dose, more than 250 mg once daily for a year, but lower doses do not have such an effect. Reports of patients with 1 g with no retinal complications have also been described. The Royal College of Ophthalmology explains the prevalence of 1.6%, which is lower than the previously reported prevalence of 7.5% by Mells and Marmor study [74].

Chloroquine maculopathy is categorized into five stages, depending on the VA and the fundus examination findings. The stages are pre-maculopathy (loss of the foveal reflex and development of fine granular changes with mild abnormalities of CV), early maculopathy (modest reduction of VA 6/9-6/12 and central foveal pigmentation surrounded by a pigmented zone of RPE atrophy giving rise to a "window" rise defect), established maculopathy (reduction of VA 6/18-6/24 and obvious bull’s eye presentation), severe maculopathy (reduced VA and RPE atrophy surrounding the fovea), and end-stage maculopathy (as per severe, additionally with arterioles and attenuated, pigmented clumps in the peripheral retina) [75].

Hydroxychloroquine is considered a safer chloroquine and since the provided dose does not exceed 400mg, the possibility of toxicity of the retina is negligible. Researchers advise this drug to be prioritized by physicians in their practice [76].

Phenothiazines: Thioridazine is used to treat schizophrenia and related psychosis. The clinical picture involves "salt-and-pepper" pigmentary disturbances, coarse plaque-like pigmentation, and focal loss of the RPE and the choriocapillaris. Chlorpromazine is used as a sedative and for schizophrenia treatment. High doses for a prolonged period may provoke granularity and clumping, as well as yellow/brown granules at the anterior lens capsule and corneal endothelial deposits [77].

Toxic crystalline maculopathies: Tamoxifen is a specific anti-estrogen used in the treatment of selective breast carcinomas. High doses of tamoxifen on the macula appear in bilateral, multiple, crystalline yellow, ring-like deposits, which persist after cessation of the treatment. Vortex keratopathy and optic neuritis can also occur but are reversible [77].

Canthaxanthin is a carotenoid used to enhance sun tanning. Over prolonged periods, it may cause tiny, glistening, yellow deposits in a symmetrical, doughnut-shaped arrangement at the posterior poles, usually located at the superficial retina [77].

Methoxyflurane is an inhalant general anesthetic. It is metabolized to oxalic acid that, in combination with calcium, forms insoluble salt calcium oxalate, which is deposited in tissues, including the RPE. Crystals within the retinal vasculature may also occur [78].

Miscellaneous Maculopathies

Idiopathic polypoidal choroidal vasculopathy: Idiopathic polypoidal choroidal vasculopathy (IPCV) is a rare abnormality of the inner choroidal vessels consisting of a dilated vascular network and multiple aneurysmal perturbances in a polypoidal shape with a predilection for the macular area. Retinal pigment epithelial detachments and a representation of choroidal neovascular membrane can also be present. The polypoidal lesions appear to be responsible for occasional leakage and hemorrhage formations under the RPE and sensory retina [79].

Prevalence of IPCV: In a retrospective study by Sho et al., taking place between 1999 and 2001, among 471 eyes that underwent a complete ophthalmologic examination and fluorescein green angiography, 110 eyes (77%) of 100 patients were diagnosed with IPCV, and the mean age was 68.4 years, with the majority being male and the involvement being unilateral (90%). The incidence of severe visual loss (0.2 or more) was 35% in IPCV and 53% in AMD. However, the above study examined solely Japanese patients, which limits the accuracy of data in regard to the ethnicity parameter [80]. The latest developmental research in this area verifies the high incidence of the condition in Asian populations in combination with early and late AMD being 1.4% to 37.9% and 0.1% to 7.3%, respectively [81].

Several hospital-based and clinical-based studies in the past decade reported a higher prevalence of IPCV in Asians. In particular, the documented proportion of IPCV in White patients ranged from 4% to 13.9%, compared to 22.3% to 61.6% reported in the Asian population [80,82]. Essentially, the accurate prevalence rate of IPCV remains unclear in the general population, as the diagnosis solely based on fundus photography is difficult. However, this is the applied technique used in the majority of epidemiological studies [83].

There is a genetic component that has been involved in the pathogenesis of IPCV in combination with AMD. This includes complement factor H (CFH) placed in the 1q32 gene and the two genes on chromosome 10q26, high-temperature-requirement-factor A1 (HTRA1), and age-related-maculopathy-susceptibility 2 (ARMS2). Considerable interactions between smoking and formyl peptide receptor 1, complete factor G, and HTRA1 genes have also been detected [84]. Additional genetic associations include missense mutation on the cholesteryl-ester-transfer protein gene, a molecule that is also involved in the metabolism of high-density lipoproteins [85].

Optic disc pit maculopathy: Optic disc pit maculopathy (ODP maculopathy) is defined by the concentration of intraretinal and subretinal fluid in the area of the macula [86]. Optic pit disc is rare, typically unilateral, but the bilateral presentation has been recorded in 15% of the ODP cases. Although ODP is abundantly a congenital anomaly, autosomal dominant inheritance has been reported in some families [87,88]. No gender predilection was noted and ODP rates an estimated prevalence of two in 10,000. Although there are studies that support the hypothesis that ODP could have an underlying genetic parameter, specific genetic associations have not been identified yet [89].

Pathophysiology of ODP maculopathy: Despite the advances in fundus photography in recent years, the exact pathogenesis and the precise origin of the fluid remain obscure. While some studies have displayed that the subretinal fluid might derive from the vitreous or the subarachnoid space, others support that the fluid is the result of the leakage from abnormal vessel formation located at the base of the pit [90,91].

A study by Brockhurst R. J. found the age of occurrence to be during the third or fourth decade of life, but many cases can occur earlier [92]. Jain et al., in their study, aimed to approach the pathogenesis of OPD and suggest that the pathology could be explained as a result of a split-like separation of the retinal layers (schisis) that communicate with the pit followed by the detachment of the outer retinal layer from the RPE [90]. On fundus examination, the condition will appear as a single grey, oval depression of the optic disc, detected most commonly at the inferotemporal segment of the disc and secondarily to the central section [93]. OPD is usually asymptomatic, with the individual not complaining of VA issues, and is diagnosed incidentally, may cause visual field defects, such as paracentral arcuate scotomas, enlarged blind spots, or mimic the clinical presentation of glaucoma [94].

Solar maculopathy: Solar maculopathy is the visual disturbance caused by the photochemical effects of solar radiation as a result of directly or indirectly looking at the sun.

Prevalence of solar maculopathy: A prospective observational case series carried out by Sohag University showed the mean age of affected individuals to be 15.5 years, with both eyes affected in four patients and the VA of the affected eye raged from 0.4 to 0.9 on presentation. Metamorphopsia was observed in 40% of individuals and central scotoma in 80% with no anterior segment anomaly and normal intraocular pressure in all cases [95].

Pathophysiology of solar maculopathy: Other symptoms included severe foveal pathology followed by circumscribed PRE mottling. Improvement of visual equity began one week after exposure and reached its maximum six months post-exposure, then became stationary. The outer retinal hole in ocular coherence tomography (OCT) persisted in 80% of cases despite good vision being restored [96-98].

**Figure 10:**
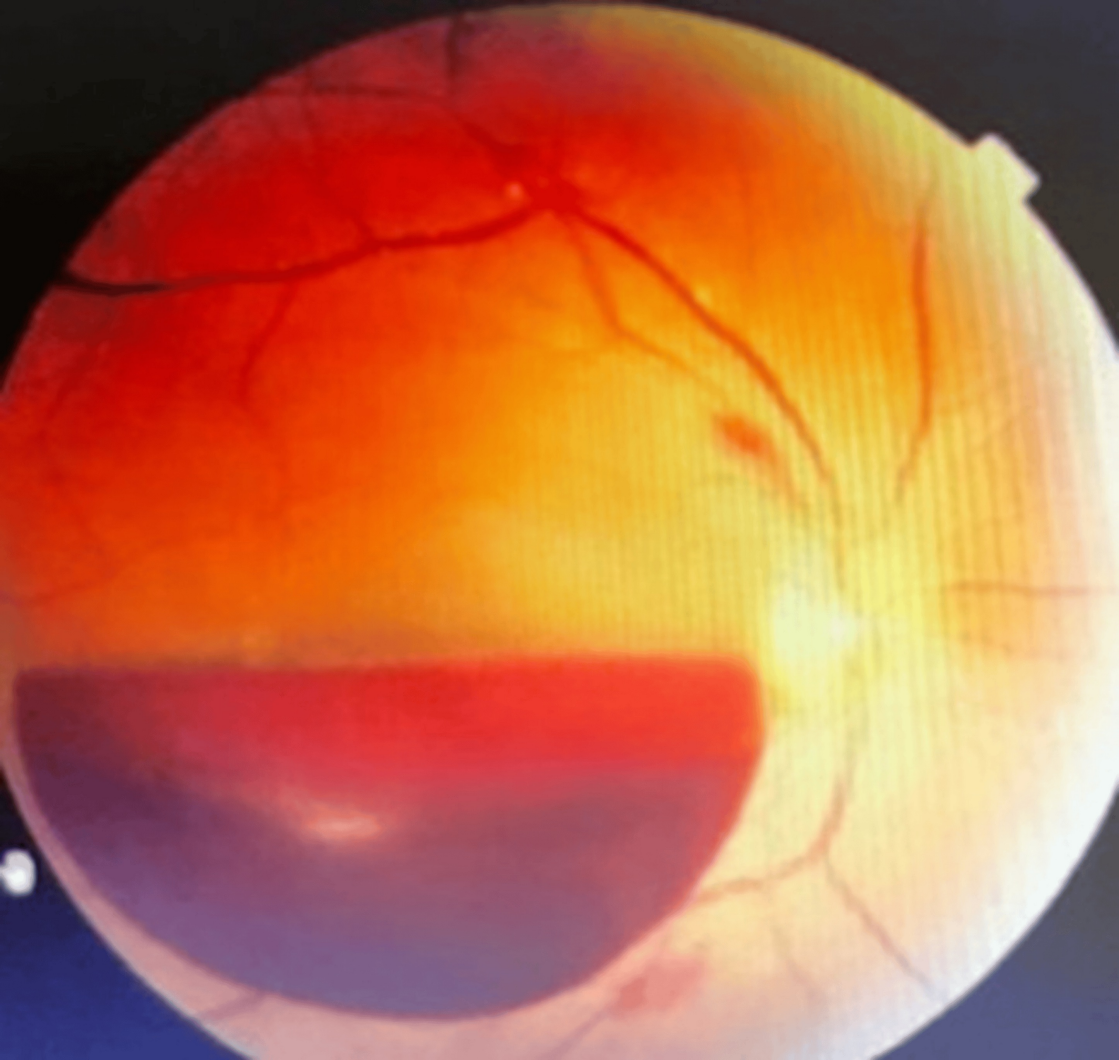
Fundus photo of the right eye illustrating the premacular sub-hyaloid and sub-inner limiting membrane (ILM) hemorrhage, diagnosed as Valsalva maculopathy Source: [99] (Permission was obtained from the original publisher)

Valsalva maculopathy: Valsalva maculopathy is a rare condition caused by an acute, severe increase in intra-abdominal or intra-thoracic pressure, resulting in intraocular bleeding (Figure 10) [99].

Pathophysiology of valsalva maculopathy: The rate of the hemorrhage settlement depends on how large and dense the hemorrhage is; spontaneous resorption without compromising the individual’s VA and with no further need for laser intervention was suggested for preretinal and subretinal membrane hemorrhages by Kumar et al.'s case series [100]. However, interventions with intravitreal tissue plasminogen activator with gas may be beneficial in cases where the hemorrhage is deeper in the retinal tissue of the eye [101-103]. Various presentations have been described, which include everyday activities, and result in Valsalva maculopathy, such as stressful aerobic exercise, inflating balloons, dental and prostate surgery, straining and constipation, vomiting, and weightlifting [104-106].

**Figure 11:**
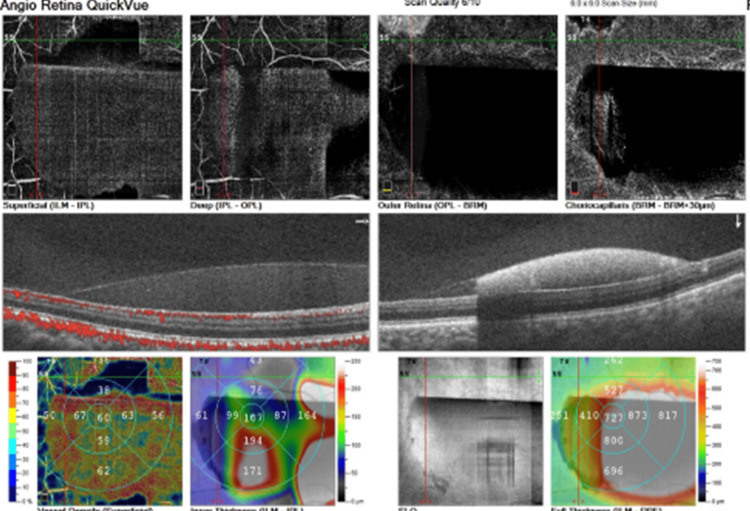
Ocular coherence tomography (OCT) and OCT angiography illustrating the premacular sub-hyaloid and sub-inner limiting membrane (ILM) hemorrhage, with Valsalva maculopathy at admission Source: [106] (Permission was obtained from the original publisher)

**Figure 12:**
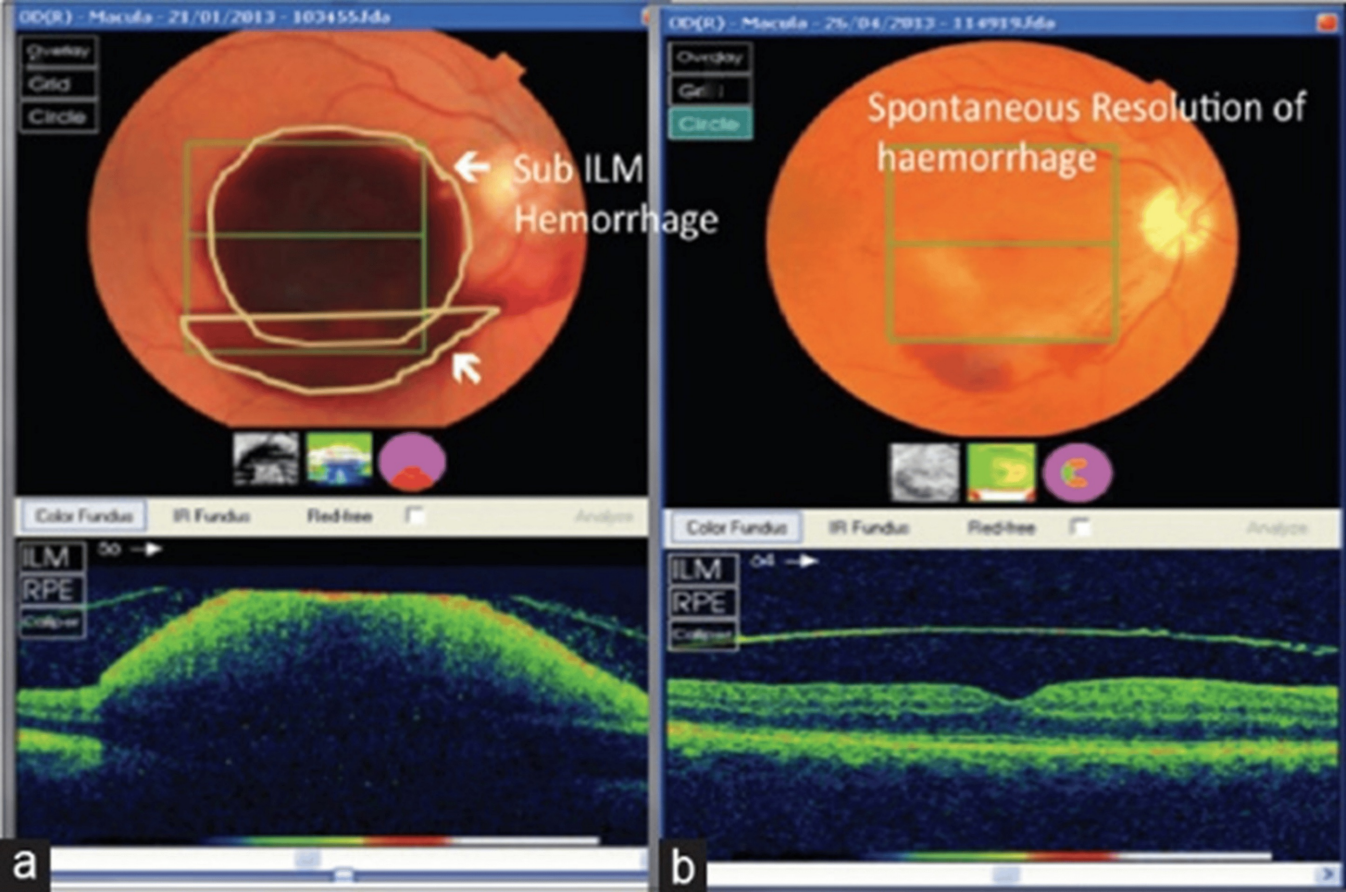
(a) Fundus photo and ocular coherence tomography (OCT) of a pre-macular sub-hyaloid hemorrhage in a patient with Valsalva maculopathy. (b) Spontaneous resolution Source: [107] (Permission was obtained from the original publisher)

A 2020 case report of a 43-year-old individual with no past medical history nor any systemic disease showed the association of a sudden, painless vision loss in the right eye after a very strong, forceful shout. Intraocular pressure remained within the normal range in both eyes (13 mmHg on the right and 14 mmHg on the left eye) and OCT angiography analysis verified the Valsalva maculopathy diagnosis. Ocular examination one week post the event showed a relative decrease in the sub-hyaloid hemorrhage but no improvement in VA. Anisometropia was also reported, and the latter improved to 20/25 at a follow-up exam, one month after the first presentation to the clinic (Figures 11-12). The above case was explained as a normal physiological response consisting of stimulation of both the sympathetic and parasympathetic branches of the autonomic nervous system (ANS) that increase the venous pressure and create a hypoxic condition in the retinal vessels. In addition, factors including higher temperature, altitude, and severe dehydration can also lead to Valsalva maculopathy [106,107].

Prevalence of valsalva maculopathy: Epidemiological records are not known, and most research papers cover individual case studies. In addition to that, there is no data in the literature, suggesting its dependence on age, sex, or racial predilection so far [101].

Public Health

**Figure 13:**
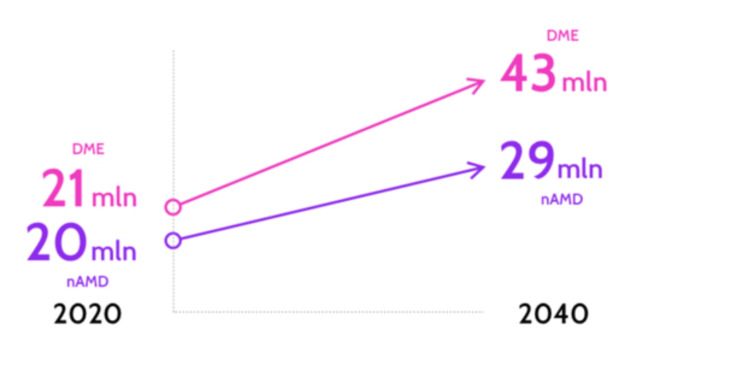
Projected number of people worldwide living with diabetic macular edema (DME) and neovascular age-related macular degeneration (AMD) Source: [108] (Permission was obtained from the original publisher)

Epidemiology: Maculopathy is a significant public health issue that is more prevalent in our societies than ever before. The number of people with a primary diagnosis of the two leading causes of vision loss, neovascular age-related macular degeneration (nAMD), and DME worldwide is already significant, and it is set to grow (Figure 13) [108,109].

AMD is a leading cause of blindness worldwide and is the most common cause of blindness in developed countries causing more than 50% of blind registration in Western societies [110].

In the UK, two in three registrations of partial or total visual loss are attributed to either wet AMD or geographic atrophy. It is calculated that more than 250,000 adults suffer from severe visual impairment because of AMD [110]. Universally, in 2020, approximately 596.2 million people have impaired vision, specifically long-sightedness, of whom 43.3 million are blind and 295.1 million have moderate or severe vision impairment (MSVI) [111].

**Figure 14:**
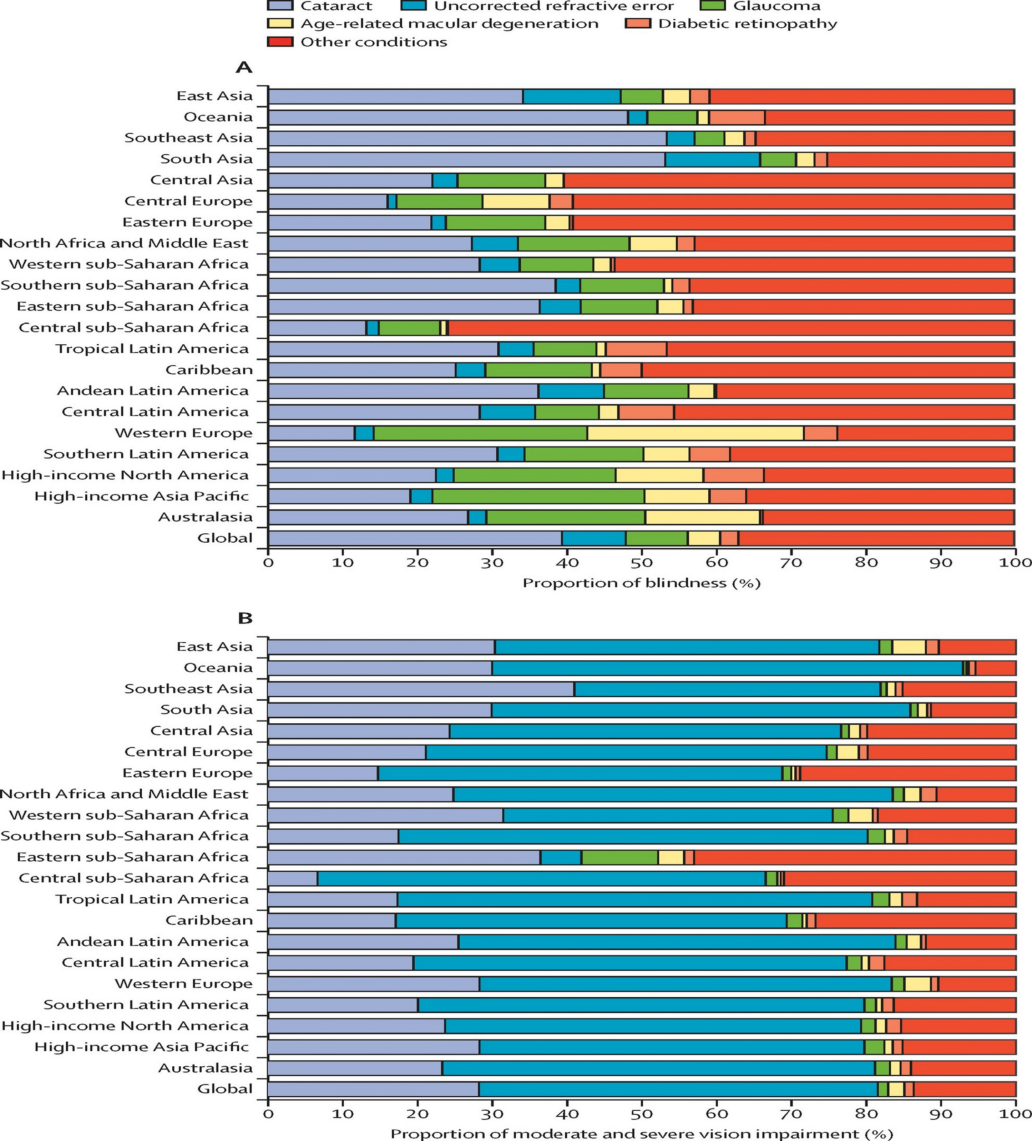
Cause of vision loss presented by the Global Burden of Disease. (A) For blindness and (B) for moderate and severe vision impairment Source: [112] (Permission was obtained from the original publisher)

**Figure 15:**
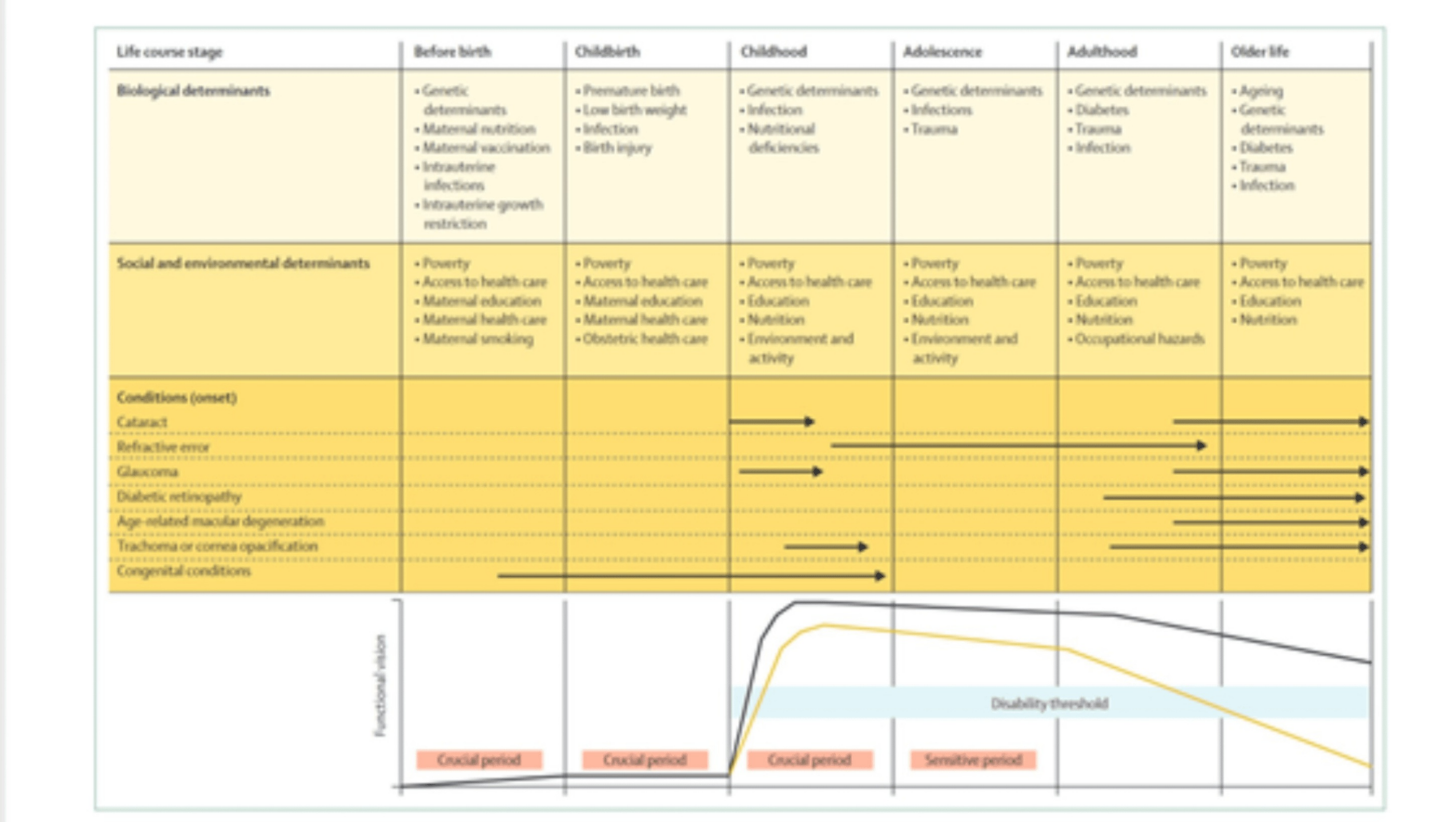
Life course perspective on eye health. Arrows indicate eye conditions and the age they occur. The yellow line indicates a hypothetical functional vision trajectory with a condition leading to increased vision impairment. The black line represents functional vision without vision impairment. The disability threshold reflects the level of functional vision below this level and is termed functional vision impairment. Source: [113] (Permission was obtained from the original publisher)

Diabetic maculopathy is one of the main preventable causes of blindness and MSVI globally (Figures 14-15), which affects adults at working age and later in life. AMD is also a leading cause of blindness and MSVI and affects mainly adults’ older lives globally [112].

Diabetic retinopathy/maculopathy remains among the commonest causes of preventable complete vision loss in most countries worldwide, and since 2014, it has been the second cause of blindness after inherited retinal diseases in both England and Wales [112,113].

While the prevalence of the disease remains high, the incidence of the disease from 2007 to 2015 in Wales has shown a tendency to reduce by almost half [114]. This trend is mainly due to the introduction of community-based diabetic retinopathy screening, resulting in the early treatment of sight-threatening DR. This underlines the importance of early diagnosis through screening policies in the community [115].

In Europe, there are over 30 million blind and partially sighted people, while an average of one in 30 Europeans experience sight loss. There are socioeconomic implications and inequality issues in the context of sex, age, and employment that relate to visually impaired individuals. The average unemployment percentage of blind and partially blind people of employment age is over 75%. Women are disproportionately affected with blindness or partial sight loss and therefore have a higher chance of being unemployed. Moreover, sight decline is proportionate to older age. One-third of citizens within the elderly population (over 65 years) experience vision loss and 90% of individuals with vision problems are over 65 years [116].

Financial implications: Maculopathy has a significant economic impact on health systems. In the US, during the year 2017, the total estimated cost of diagnosed diabetes was $327 billion, of which $237 billion was attributed to direct medical costs and $90 billion to reduced productivity. Of these costs, 25% was attributed to morbidity related to the eyes [117].

Moreover, AMD management has a significant socioeconomic impact on health systems. In the UK NHS hospitals, there has been a significant increase in hospital burden related to the treatment and monitoring of people with AMD; in the years 2005−2006, less than 10,000 visits were recorded but increased to over 75,000 visits in 2013−2014 (Hospital Episode Statistics) [111]. In the UK, the most commonly used anti-VEGF medications are aflibercept and ranibizumab. The cost of this repetitive treatment is significant; in 2015−2016, ranibizumab was second and aflibercept was fourth on the list of medicines with positive NICE technology appraisals on which the NHS spent the most, the total cost of both amounting to around £450 million (NHS Digital, 2016) [110]. In the US annual Medicare Part B, the spending for anti-VEGF treatments in the year 2015 was $2.96 billion, while the average cost for each patient receiving anti-VEGF injections was approximately $9,820 yearly [111,118].

Diabetes accounts for significant healthcare costs, productivity decline, mortality at a premature age, and, overall, a low standard of living. In particular, in the US, in 2012, the financial implications associated with diabetes diagnosis were calculated to be $245 billion in the form of higher medical costs ($176 billion) and reduced productivity ($69 billion). It has been estimated that in 2017, one in every seven healthcare dollars (14%) is attributed to diabetes. Interestingly, healthcare expenditures attributed to diabetes are considered to be the additional expenditures the nation incurs because of the condition. This equates to the total healthcare expenditures for people with diabetes minus the projected level of expenditures that would have occurred for those in the absence of the disease. In 2017, the total expenditure of the US for the health cost components included accounting for nearly $1.7 trillion in projected expenditure.

**Figure 16:**
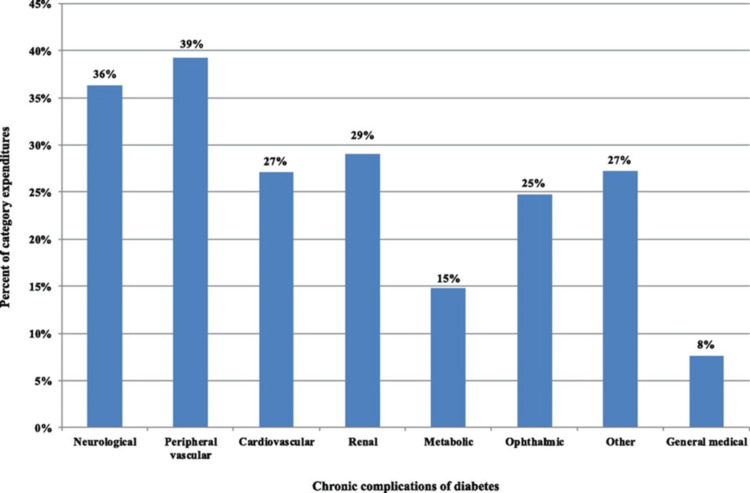
Percentage of medical condition-specific expenditures associated with diabetes Source: [117] (Permission was obtained from the original publisher)

Approximately $414 billion (almost 24%) of the total is incurred by patients with diabetes. Costs exceed $237 billion, which equals 57% of the total medical costs attributed to diabetes diagnoses [117]. Since ophthalmic diabetic-related conditions like diabetic maculopathy constitute 25% of the total expenditures of diabetes care (Figure 16), it becomes evident that DR is a very important contributor to medical care costs.

**Figure 17:**
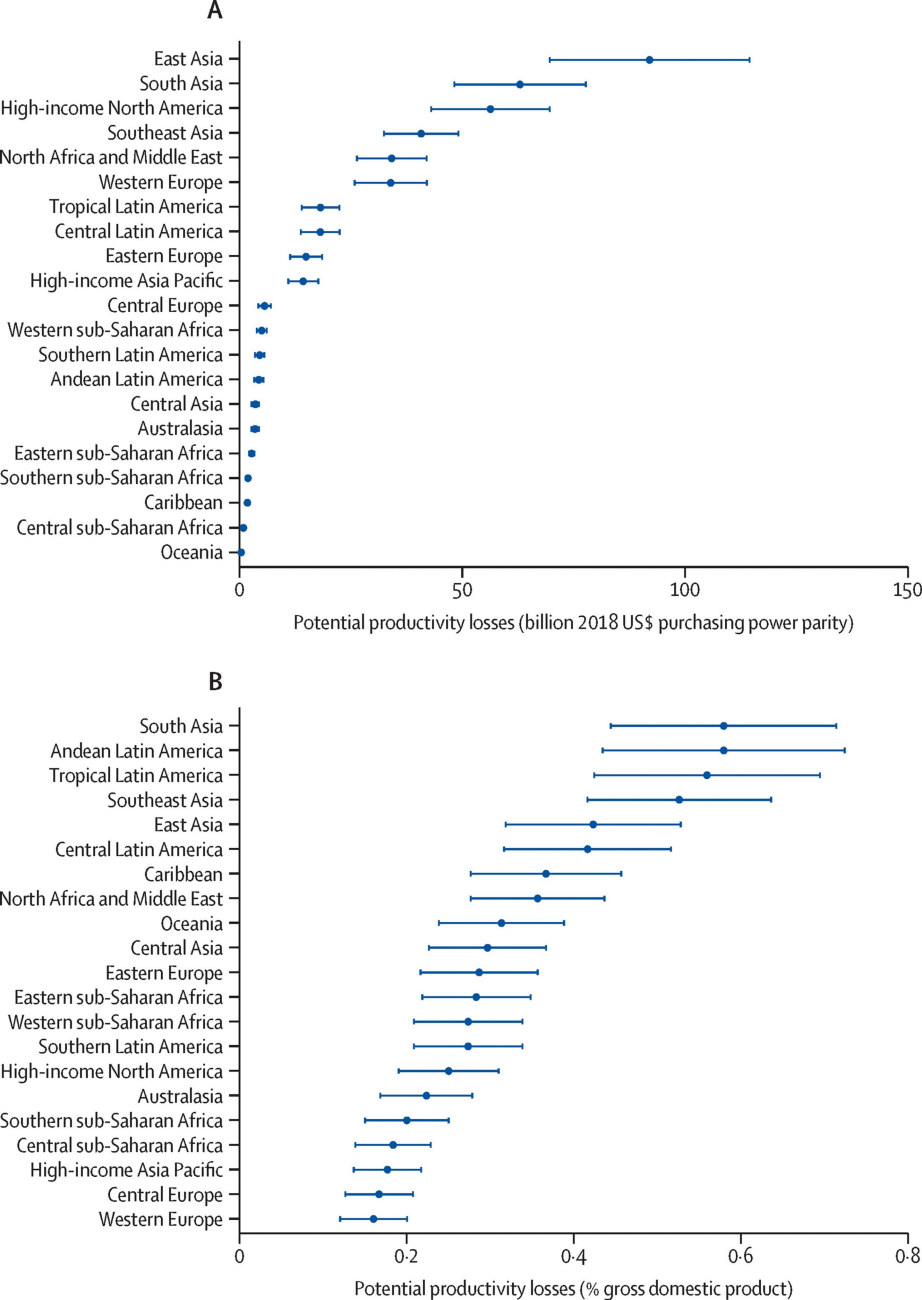
Global productivity loss estimates due to blindness and moderate or severe vision impairment (MSVI) derived from the gross national income in the 21 Global Burden of Disease regions in 2018 (A) in billion USD purchasing power parity (PPP) and (B) as a percentage of gross domestic product (GDP) Source: [96] (Permission was obtained from the original publisher)

In 2020, the overall relative increase in unemployed individuals with complete sight loss or MSVI was recorded to be 30.2%. It is reported that the international annual productivity loss was $410,7 billion, which is 0.3% of the gross domestic product (GDP) of the 21 Global Burdens of Disease (GBD) Study regions in 2018. Potential productivity losses related to blindness were $43.6 billion and $367.1 billion related to MSVI. Figure 17 shows the productivity losses for each GBD region [96].

Psychosocial implications: Vision impairment affects multiple functional domains (physical, psychological, social), and the overall individual’s wellbeing [119]. It has been noticed that depression and anxiety, expressed mainly as agoraphobia and social phobia, were the major public health problems in visually impaired older adults [120]. Additionally, individuals with severe unilateral and bilateral vision impairment when compared to those with no visual impairment are significantly associated with worse vision-specific functioning, regardless of the level of education and literacy adequacy. This suggests that visual impairment affects people independent of socioeconomic factors [121].

In children, ocular disorders that occur in infants, toddlers, and young adolescents usually present as lifelong problems from puberty to adulthood. When significant vision impairment is present in a child, there are important effects on their future developmental portfolio and overall health, such as self-perception, educational attainment, job prospects, and a number of other evolvement and adaptation factors [122].

**Figure 18:**
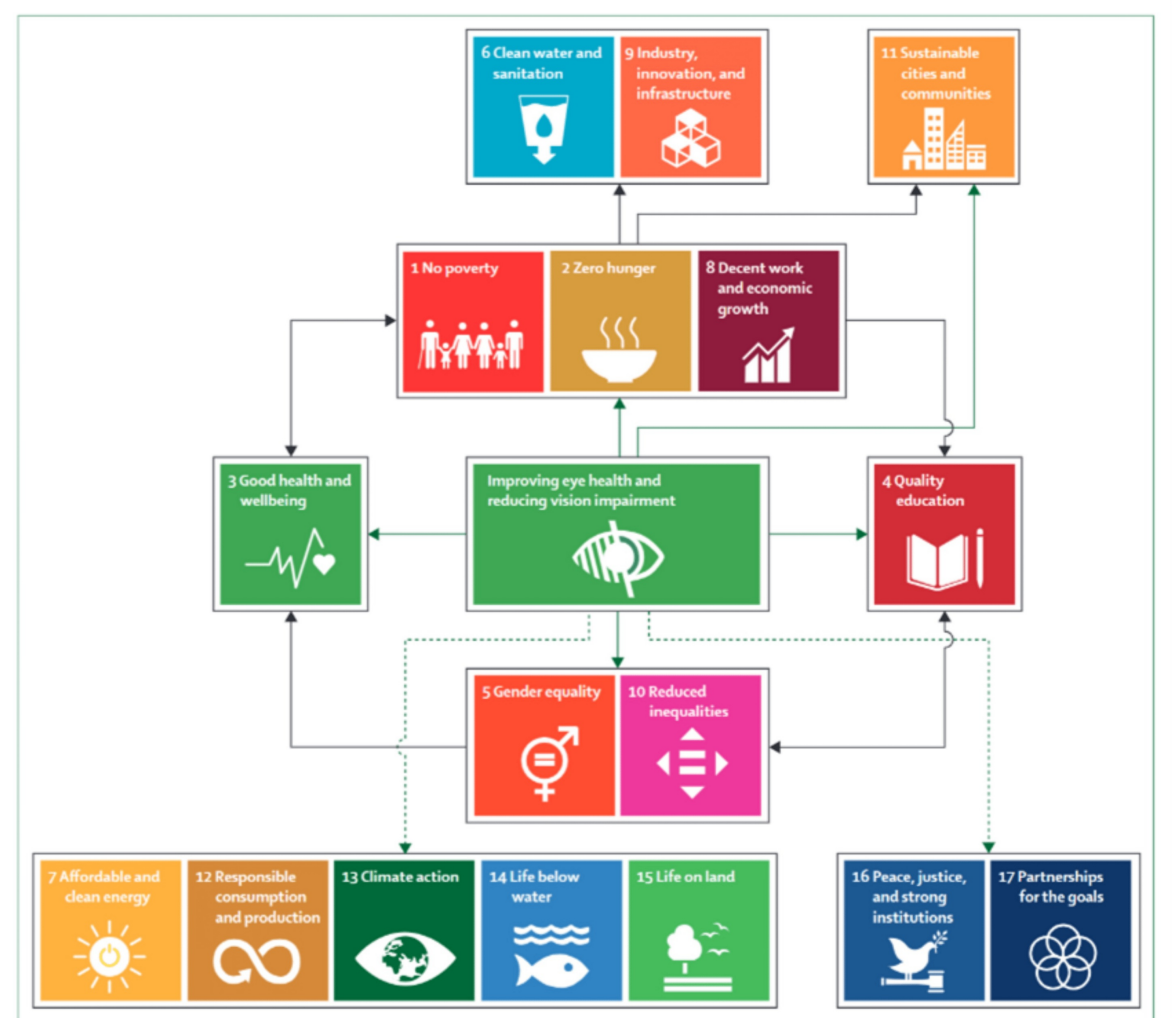
Improving eye health and Sustainable Development Goals. Green arrows: direct evidence of beneficial effect. Dashed arrows: hypothesized direct beneficial effect. Black arrows: possible indirect beneficial effects. Source: [112] (Permission was obtained from the original publisher)

The vision as an issue of sustainable development: The UN SDGs (Sustainable Development Goals) are a group of broad target-driven goals for 2030, and they are designed as a “blueprint to achieve a better and more sustainable future for all” [123]. Vision contributes to multiple everyday life activities, supports high-quality education, and enhances productivity, improving the overall quality of society. A recent study (based on cataract and refractive error-related visual impairment) has shown that vision has the potential to support the SDGs, by contributing toward poverty reduction, zero hunger, good health, and wellbeing (Figure 18). For this reason, eye health is projected as a global public priority, which has a determinant role in transforming directly and indirectly the lives globally and can therefore be considered a broad-based sustainable development issue [112].

Current and Novel Treatments

Therapeutic options in MDs: A hallmark of MDs is symmetrical central vision loss. In the past decade, the molecular basis of this heterogeneous group of disorders has been investigated with advancements in genetic testing. However, there is currently no definite treatment for these conditions that benefit both the vision and the quality of living. In this section, the treatment modalities for STGD and Best disease are discussed. The novel treatment options are currently either in clinical trials or under future research planning [15].

STGD: As in all MD patients, patients with STGD are provided with vision aids and assistive techniques to facilitate their sight. They also offered appropriate social support and advice on a healthy diet and daily activities to potentially achieve a slower progression of the condition. Emphasis is made not to take vitamin A supplements and to reduce UV ray exposure with everyday adaptations, eg., wearing sunglasses and avoiding cigarette smoking [124].

In addition, medications that target either directly or indirectly the visual cycle have been developed. This includes the complement-mediated response to accumulated by-products of the visual cycle. Drugs, such as soraprazan, emixustat, STG-001, and fenretinide, impede the formation of A2E (a component of ocular lipofuscin) and lipofuscin, acting as visual cycle modulators via different biochemical mechanisms. Most of these modulators have gone through phase I/II or III of clinical trials. The antioxidant supplement saffron for the treatment of STGD is also in phase II clinical trial and has proved to have a good safety profile [124].

Gene replacement in preclinical studies showed improvement of the phenotypes in ABCA4−/ − mice and have supported the development of clinical trials regarding gene therapy in human subjects [125]. Stem cell-based strategies are also being investigated. One phase I/II clinical trial of human embryonic stem (hESC)-derived RPE cells in STGD that has been completed, has shown no convincing results of functional visual improvement 12 months post-treatment. New approaches to combining RPE and photoreceptor cell transplantation are under evolvement for the treatment of STGD [126].

Best's disease: Prognosis may often be relatively good in Best's disease compared to other MDs. However, gradual resorption of subretinal substances can link to visual deterioration. Best's disease can be perplexed by neovascular tissue growth, which can result in severe, acute vision deterioration. In the case of the development of CNV, one or two intravitreal injections of bevacizumab (Avastin) are found to effectively improve both structural and functional conditions within the macula [127].

Diabetic maculopathy: Diabetes mellitus is a growing epidemic in regard to prevalence and has significant morbidity and mortality rates globally [115]. DME is a leading cause of visual impairment among patients with eye conditions and poses a substantial challenge to healthcare systems [128]. Thus, the need for prompt treatment poses a challenge on a personal, social, and financial level globally.

**Figure 19:**
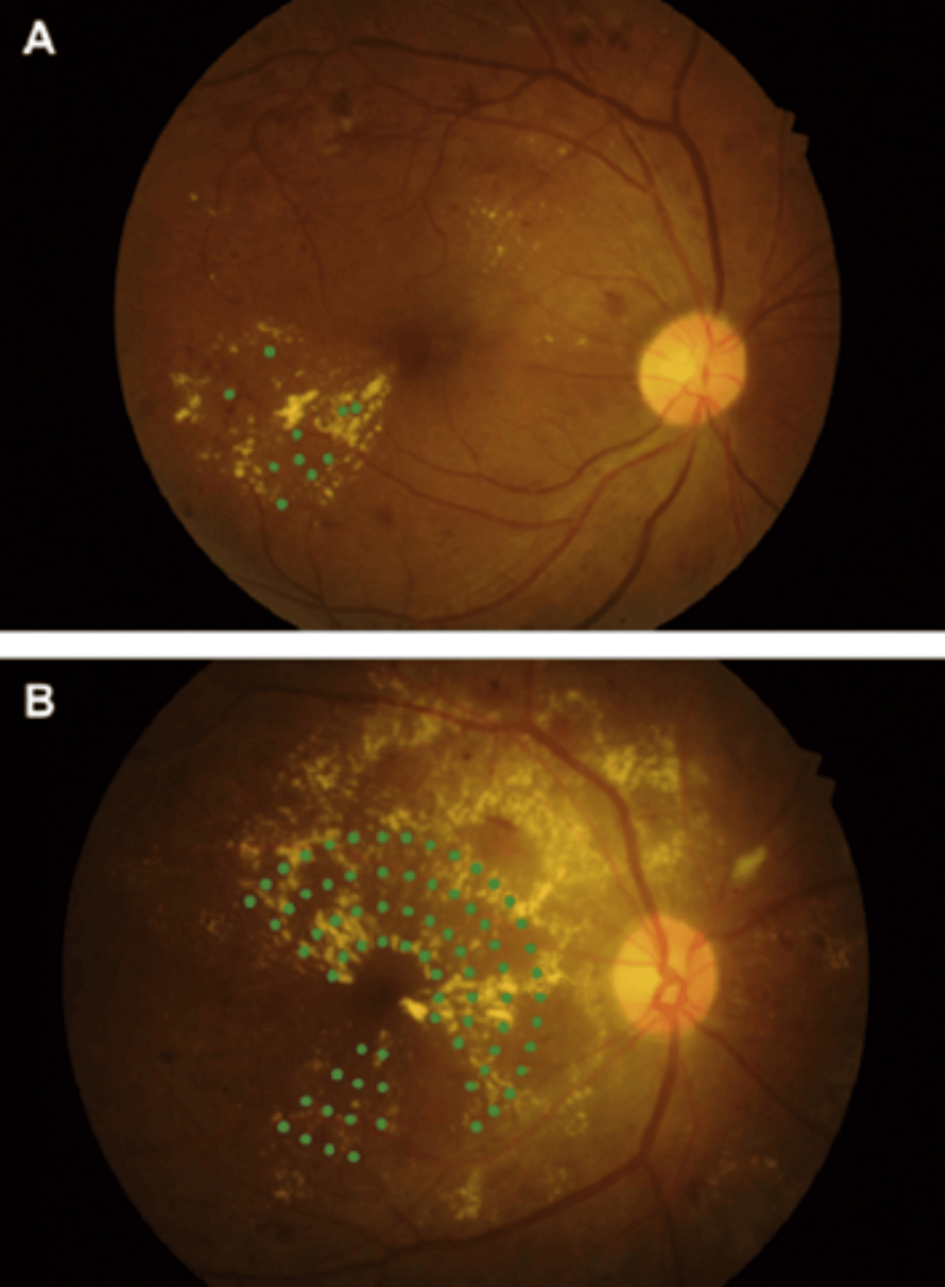
(A) Focal and (B) modified grid laser patterns in the treatment of diabetic macular edema Source: [131] (Permission was obtained from the original publisher)

Diabetic control with the marker HbA1c less than 7%, treatment of systemic hypertension, and hyperlipidemia are related to reduced prevalence of DR among diabetics [129,130]. Focal/grid or modified grid laser (Figure 19) was once the only available treatment for the DME [131]. Now, it is reserved only for non-center involving macular edema (NCI-DME, which does not present within 500 microns from the center of the fovea) either alone or in combination with anti-VEGF injections.

Today, anti-VEGF therapy protocols have been developed and are the mainstream treatment of DME. Intravitreal injections of long-lasting, slow-release corticosteroids can be effective in treating DME adjunctively, but adverse effects such as intraocular pressure elevation and cataract development must be considered and therefore closely monitored [132].

In Table 3, the combined treatment modalities for DME (and diabetic retinopathy) are summarized through the Preferred Practice Patterns (PPP) by the American Academy of Ophthalmology [133].

**Table 3:** Initial management recommendations for patients with diabetes. Anti-VEGF: anti-vascular endothelial growth factor; CI-DME: center-involved diabetic macular edema; NCI­ DME: noncenter-involved diabetic macular edema; NPDR: non-proliferative diabetic retinopathy; PDR: proliferative diabetic retinopathy * Adjunctive treatments that may be considered include intravitreal corticosteroids or anti-VEGF agents (off­-label use, except aflibercept and ranibizumab). ' For patients with good visual acuity (20/25 or better) and CI-DME, there is no difference between observation plus aflibercept if visual acuity decreases, focal laser plus aflibercept if visual acuity decreases, or anti-VEGF treatment. It is appropriate to defer treatment until visual acuity is worse than 20/25. Exceptions include hypertension or fluid retention associated with heart failure, renal failure, pregnancy, or any other causes that may aggravate macular edema. Deferral of photocoagulation for a brief period of medical treatment may be considered in these cases. Moreover, deferral of NCI-DME treatment is an option if visual acuity is excellent (better than 20/32), close follow-up is possible, and the patient understands the risks [134]. (This is an open access Table from the Preferred Practice Patterns by the AAO.)

Severity of retinopathy	Presence of macular edema	Follow-up (months)	Pan-retinal photocoagulation (scatter) laser	Focal and/or grid laser*	lntra-vitreal anti-VEGF therapy
Normal or minimal NPDR	No	12	No	No	No
Mild NPDR	No	12	No	No	No
	NCI-DME	3-6	No	Sometimes	No
	CI-DME'	1*	No	Rarely	Usually
Moderate NPDR	No	6-121	No	No	No
	NCI-DME	3-6	No	Sometimes	Rarely
	CI-DME'	1*	No	Rarely	Usually
Severe NPDR	No	3-4	Sometimes	No	Sometimes
	NCI-DME	2-4	Sometimes	Sometimes	Sometimes
	CI-DME'	1*	Sometimes	Rarely	Usually
Non-high-risk PDR	No	3-4	Sometimes	No	Sometimes
	NCI-DME	2-4	Sometimes	Sometimes	Sometimes
	CI-DME'	1*	Sometimes	Sometimes	Usually
High-risk PDR	No	2-4	Recommended	No	Sometimes
	NCI-DME	2-4	Recommended	Sometimes	Sometimes
	CI-DME'	1*	Recommended	Sometimes	Usually

Age-related maculopathy: The management of AMD depends on the type and severity of the disease, involving measures for early detection and treatment. Healthy lifestyle adaptations and predominantly smoking cessation are recommended to patients in all stages of AMD since further degeneration can be delayed with such changes. Dietary supplementation is advisable in the intermediate severity of all types of AMD, but has an inferior effect compared to smoking cessation alone and provides no protection in the early stages of AMD. In addition, no effect on late stages of either dry or wet AMD has been reported [134].

While patients with dry AMD are still hoping for the development of an efficacious treatment, patients with wet AMD have a variety of effective anti-VEGF medications available even though some of them have suboptimal responses or compliance is reduced [19]. After the establishment of the diagnosis and the stage of AMD, patients will need to be provided with a customized approach tailored to their personal condition, which includes (a) information on the current stage and future progress of their condition, (b) consultation on lifestyle and dietary issues (including smoking cessation, diet improvement, exercise initiatives, and weight loss), (c) advice on vision standards and driving, (d) screening tests establishment and follow-up, and (e) treatment options, benefits, and risks (mainly endophthalmitis).

Additional advice on approved dietary supplements, continuous comorbidity assessment, and awareness of the psychological impact (e.g., depression) such conditions may have are all vital in AMD management. Easily accessible information, leaflets, and local national support groups with shared experiences and a clear direction into the management of AMD must be taken into account.

**Figure 20:**
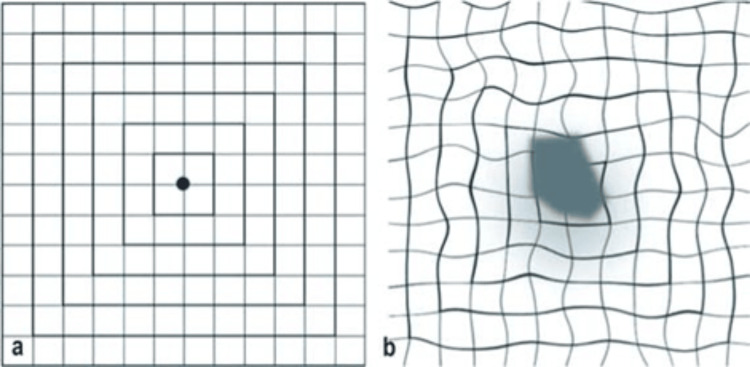
Amsler test: (A) normal macular findings with the vertical and horizontal lines being straight. (B) Late AMD: the central fixation point cannot be seen because of the central scotoma. The vertical and horizontal lines appear distorted (metamorphopsia). Source: [135] (Permission was obtained from the original publisher)

Early diagnosis measures with the use of the Amsler grid chart are presented in Figure 20 [135].

The main part of the management of early and late (either dry or wet inactive) AMD involves self-monitoring. AMD patients need to understand the importance of early diagnosis and the role of self-monitoring in early diagnosis. Regular appointments to check their vision at the community optometrists are advised, as well as active monitoring and reporting as soon as possible for any new changes such as blurred vision, new distortion of straight lines at the Amsler chart, or a change in the size of objects. The involvement of a family member or a carer is beneficial for people who may need support to reliably monitor their vision [110].

**Figure 21:**
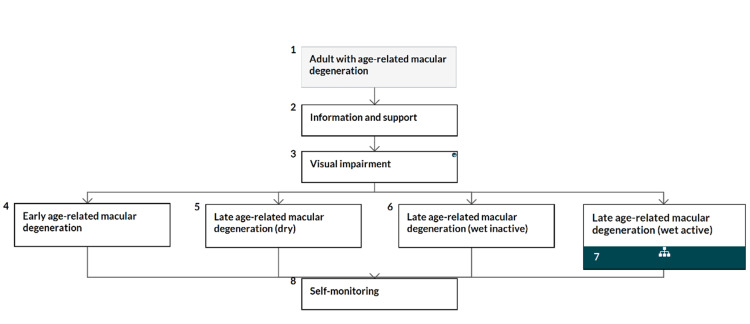
A synopsis set by the National Institute for Health and Care Excellence (NICE) for the management of all types of age-related macular degeneration (AMD) (except the wet active AMD) Source: [136] (Permission was obtained from the original publisher)

A synopsis of the National Institute for Health and Care Excellence (NICE) guidelines for the management of all stages of AMD (except the AMD wet active) is presented in the following flow chart (Figure 21) [136].

Repeated intravitreal injections of anti-VEGF factors constitute the most effective current treatment modality of active wet AMD. It has been found that they can reduce the risk of visual loss of 15 or more letters in the ETDRS chart in patients with neovascular AMD [137].

There are four existing drugs currently, one of which is off-label (bevacizumab, the first one used since 2005) and three that have been approved by the EMA and FDA: ranibizumab (approved 2007), aflibercept (approved 2012), and brolucizumab (approved 2020) [135].

The four anti-VEGF factors differ from one another, in their chemical structure, binding affinity, and specificity; however, they share the same mechanism of action, by blocking the VEGF. VEGF has a dual action; it is pro-angiogenic, promoting the formation and growth of abnormal blood vessels in exudative AMD, and a facilitator of the permeability of blood vessels, which leak plasma components either into the retinal parenchyma (intra-retinal fluid) or below the neurosensory retina (subretinal fluid). The accumulation of fluid from hyperpermeable leaking choroidal vessels is one of the main causes of vision deterioration in active exudative AMD. The main action against wet MD comes from the reduction of vessel permeability rather than from the inhibition of angiogenesis [138-139], while new treatment modalities aim to use stem cells to heal the impaired RPE [140].

Currently, there are two different protocols with similar effects that have replaced the initial study designs of one intravitreal injection every month: the “Per Required Need” (PRN) protocol and the “treat and extend” protocol. Most patients require seven to eight injections in the first year of treatment to control exudative AMD effectively, with fewer injections generally being needed in later years [138,140,141].

**Figure 22:**
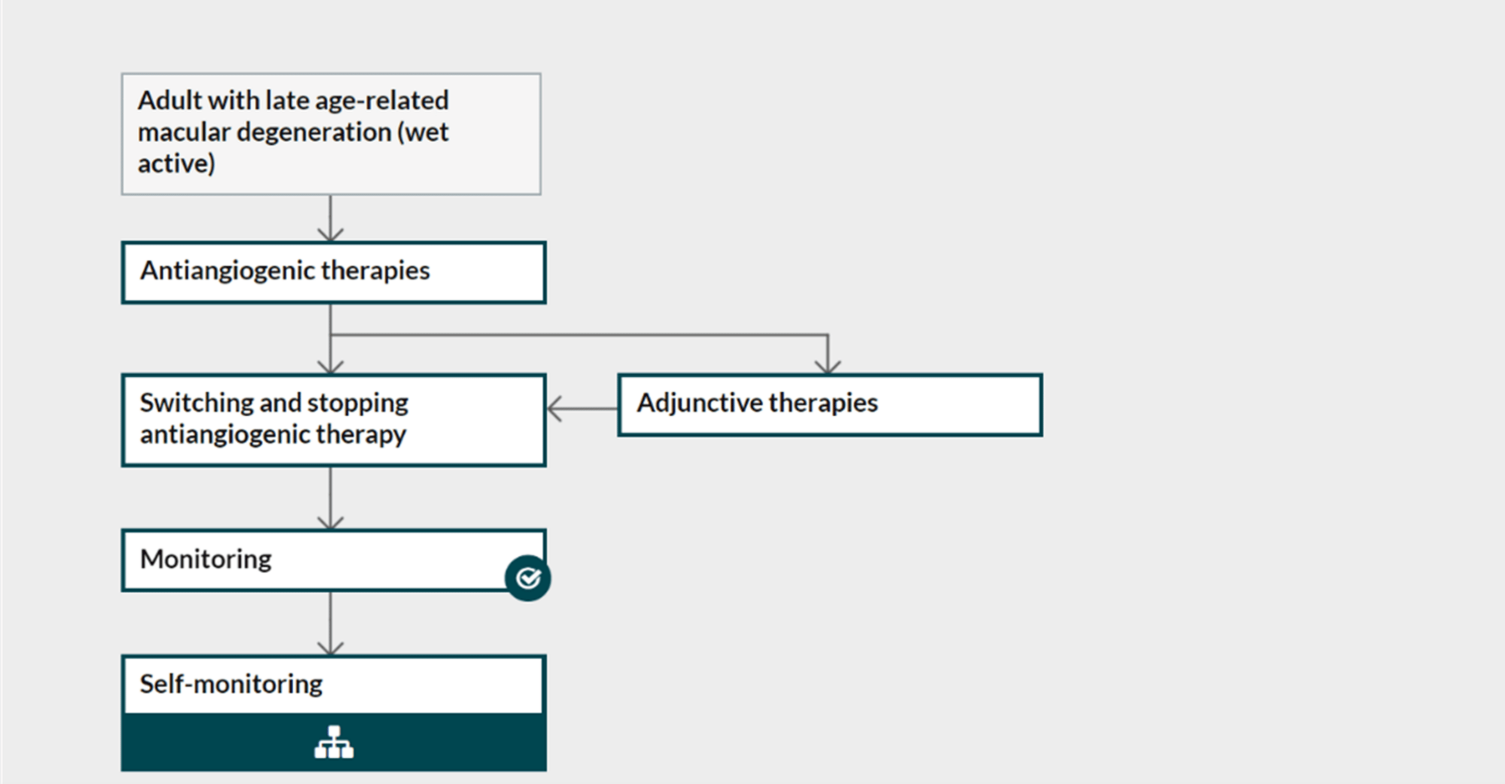
National Institute for Health and Care Excellence (NICE) treatment pathway for wet active age-related macular degeneration (AMD) Source: [142] (Permission was obtained from the original publisher)

In Figure 22, the NICE summarizes the treatment pattern for wet active AMD in a flow chart. Currently, adjunctive therapies in the form of photodynamic therapy (PDT) are not recommended for any type of AMD except in the context of clinical research [142].

Discussion

MDs represent a collection of inherited disorders of the retina that result in sight impairment, most commonly by causing progressive atrophy in the macular area of the eye. They are often characterized by bilateral, relatively symmetrical macular abnormalities affecting central vision. Scientific knowledge of MDs has made notable progress over the previous years, offering early detection, targeted advice on the progress of the condition, and newly attempted therapeutic prospects. These developments have been founded upon the availability of high-resolution multimodal imaging, advanced genetic understanding, and testing availability.

As a result, emerging therapies, either alone or in combination, are being investigated and are expected to further expand on novel treatment options. These multiple therapeutic approaches are already being tested in STGD, but fewer data have been made available for disorders like Best’s disease, which together with pattern dystrophy disease account for a significant percentage of vision loss cases. This is possibly explained by the difficulty of developing treatments that target conditions with genetic underlying etiology. However, there is hope that the research committee will overcome those challenges in the future with the focus shifting toward advancements regarding gene silencing/editing. With gene therapy in phase III trials (e.g., NCT00999609 for RPE65-LCA) and with stem cell-derived RPE transplantation in phase II trials (e.g., NCT01345006 for STGD), it is expected that gene and stem-cell therapies will be the next new treatment to become available for macular inherited degenerative diseases.

The rapid rise in diabetic cases and the increase in population age at which DME and AMD present result in growing public health responsibility for treatment requirements and high financial costs to clinicians, multidisciplinary teams, and healthcare systems worldwide. The establishment of intravitreal application of anti-VEGF injections has considerably altered the standard approach plan for the disease. In particular, there have been landmark trials showing the efficiency traits of these agents over past laser-based treatments. However, the best outcomes require frequent intravitreal injections, causing treatment fatigue, potentially leading to suboptimal management, difficulties with re-examination appointments, and, to a certain extent, irreversible vision problems. New therapeutic modalities are being investigated to reduce the frequency and improve the anti-VEFG efficacy, particularly for AMD and DME management. The introduction of brolucizumab with its anatomical and functional efficacy and the durability of its drying ability, which is to be expected due to its small molecular size that allows for a higher agent concentration and a better penetration rate, is exciting for the field. It may meet an important need not only by preserving vision but also by facilitating patients’ ability to manage other (age or diabetes or a combination of both) health comorbidities.

The UN has decided to set SDGs for 2030. In this literature review, it has become evident that there is published evidence on how working toward advancing ophthalmological healthcare can have an impactful contribution to improving the SDGs. The benefits as a result of providing and improving eye health services are evident and are related to the following SDGs: low income, education, fairness, and sustainable cities. Improving vision through upgrading the management of maculopathies is expected to contribute to a sustainable expansion of the world and the quality of vision globally.

## Conclusions

Maculopathies are a diverse family of ophthalmic diseases that present with a variety of clinical appearance and pathophysiological changes in the macular area and affect both children and adults at working and older age. They are causing severe and potentially irreversible central vision loss with a significant impact on patients’ physical, mental, psychosocial, and economic status. While the current treatment of inherited MDs is limited to vision aids, in diabetic maculopathy and age-related macular disease, intravitreal injection protocols can preserve vision for a limited period. Attention should, therefore, be paid to novel treatment research based on gene therapy and stem cells that have shown a good safety profile and given hope for future therapy. There is, therefore, an immense need to invest in health policies for the early detection and improvement of current therapeutic protocols in order to limit as much as possible any avoidable vision loss. Early diagnosis, appropriate treatment, and continuous research could together enhance well-being, overall health, and community inclusion on a personal and global level and can contribute to achieving important SDGs.

## References

[1] Rein DB, Wittenborn JS, Zhang X, Honeycutt AA, Lesesne SB, Saaddine J (2009). Forecasting age-related macular degeneration through the year 2050: the potential impact of new treatments. Arch Ophthalmol.

[2] Zhou M, Duan PC, Liang JH, Zhang XF, Pan CW (202112022). Geographic distributions of age-related macular degeneration incidence: a systematic review and meta-analysis. Br J Ophthalmol.

[3] Bandello F, Battista M, Brambati M, Starace V, Arrigo A, Parodi MB (2020). Recent developments in maculopathy. Current Concepts in Ophthalmology.

[4] (2022). Best vitelliform macular dystrophy - NORD (National Organization for Rare Disorders). https://rarediseases.org/rare-diseases/best-vitelliform-macular-dystrophy/.

[5] Altschwager P, Ambrosio L, Swanson EA, Moskowitz A, Fulton AB (2017). Juvenile macular degenerations. Semin Pediatr Neurol.

[6] Kanski J, Bowling B (2007). Clinical ophthalmology: a systematic approach. Clinical Ophthalmology: A Systematic Approach. 6th ed. Butterworth-Heinemann.

[7] (2021). Causes of blindness and vision impairment in 2020 and trends over 30 years, and prevalence of avoidable blindness in relation to VISION 2020: the Right to Sight: an analysis for the Global Burden of Disease Study. Lancet Glob Health.

[8] Geerlings MJ, de Jong EK, den Hollander AI (2017). The complement system in age-related macular degeneration: a review of rare genetic variants and implications for personalized treatment. Mol Immunol.

[9] Browning DJ, Fraser CM, Clark S (2008). The relationship of macular thickness to clinically graded diabetic retinopathy severity in eyes without clinically detected diabetic macular edema. Ophthalmology.

[10] Recchia FM, Ruby AJ, Carvalho Recchia CA (2005). Pars plana vitrectomy with removal of the internal limiting membrane in the treatment of persistent diabetic macular edema. Am J Ophthalmol.

[11] Maturi RK, Glassman AR, Liu D, Beck RW, Bhavsar AR, Bressler NM (201812022). Effect of adding dexamethasone to continued ranibizumab treatment in patients with persistent diabetic macular edema: a DRCR network phase 2 randomized clinical trial. JAMA Ophthalmol.

[12] Bailey CC, Sparrow JM, Grey RH, Cheng H (1998). The national diabetic retinopathy laser treatment audit. I. Maculopathy. Eye (Lond).

[13] Wells JA, Glassman AR, Ayala AR (2015). Aflibercept, bevacizumab, or ranibizumab for diabetic macular edema. N Engl J Med.

[14] Michaelides M, Hunt DM, Moore AT (2003). The genetics of inherited macular dystrophies. J Med Genet.

[15] Rahman N, Georgiou M, Khan KN, Michaelides M (2020). Macular dystrophies: clinical and imaging features, molecular genetics and therapeutic options. Br J Ophthalmol.

[16] Scholl HP, Strauss RW, Singh MS, Dalkara D, Roska B, Picaud S, Sahel JA (2016). Emerging therapies for inherited retinal degeneration. Sci Transl Med.

[17] Mitchell P, Liew G, Gopinath B, Wong TY (2018). Age-related macular degeneration. Lancet.

[18] (2023). Age-related macular degeneration | NICE. https://www.nice.org.uk/about/what-we-do/research-and-development/research-recommendations/NG82/1.

[19] Ammar MJ, Hsu J, Chiang A, Ho AC, Regillo CD (2020). Age-related macular degeneration therapy: a review. Curr Opin Ophthalmol.

[20] Wong WL, Su X, Li X, Cheung CMG, Klein R, Cheng CY (20141). Global prevalence of age-related macular degeneration and disease burden projection for 2020 and 2040: a systematic review and meta-analysis. Lancet Glob Health.

[21] Rozet JM, Gerber S, Ducroq D, Hamel C, Dufier JL, Kaplan J (2005). Hereditary macular dystrophies [Article in French]. J Fr Ophtalmol.

[22] Fishman GA, Farber M, Patel BS, Derlacki DJ (1987). Visual acuity loss in patents with Stargardt's macular dystrophy. Ophthalmology.

[23] Von Ruickmann A, Fitzke FW, Bird AC (199512021). Distribution of fundus autofluorescence with a scanning laser ophthalmoscope. Br J Ophthalmol.

[24] Fish G, Grey R, Sehmi KS, Bird AC (1981). The dark choroid in posterior retinal dystrophies. Br J Ophthalmol.

[25] Fishman GA (1976). Fundus flavimaculatus. A clinical classification. Arch Ophthalmol.

[26] Dorey CK, Wu G, Ebenstein D, Garsd A, Weiter JJ (1989). Cell loss in the aging retina. Relationship to lipofuscin accumulation and macular degeneration. Invest Ophthalmol Vis Sci.

[27] von Rückmann A, Fitzke FW, Bird AC (1999). Distribution of pigment epithelium autofluorescence in retinal disease state recorded in vivo and its change over time. Graefes Arch Clin Exp Ophthalmol.

[28] Von Rückmann A, Fitzke FW, Bird AC (1997). In vivo fundus autofluorescence in macular dystrophies. Arch Ophthalmol.

[29] Kretschmann U, Seeliger MW, Ruether K, Usui T, Apfelstedt-Sylla E, Zrenner E (1998). Multifocal electroretinography in patients with Stargardt's macular dystrophy. Br J Ophthalmol.

[30] (2021). Stargardt disease | National Eye Institute. https://www.nei.nih.gov/learn-about-eye-health/eye-conditions-and-diseases/stargardt-disease.

[31] (2024). Diagnosis and management of Stargardt disease. https://www.aao.org/eyenet/article/diagnosis-management-of-stargardt-disease.

[32] Lois N, Holder GE, Fitzke FW, Plant C, Bird AC (1999). Intrafamilial variation of phenotype in Stargardt macular dystrophy-Fundus flavimaculatus. Invest Ophthalmol Vis Sci.

[33] Kaplan J, Gerber S, Larget-Piet D (1993). A gene for Stargardt's disease (fundus flavimaculatus) maps to the short arm of chromosome 1. Nat Genet.

[34] Noble KG, Carr RE (1979). Stargardt’s disease and fundus flavimaculatus. Arch Ophthalmol.

[35] Collison FT, Lee W, Fishman GA, Park JC, Zernant J, McAnany JJ, Allikmets R (2019). Clinical characterization of Stargardt disease patients with the p.N1868I ABCA4 mutation. Retina.

[36] Lois N, Holder GE, Bunce C, Fitzke FW, Bird AC (2001). Phenotypic subtypes of Stargardt macular dystrophy-fundus flavimaculatus. Arch Ophthalmol.

[37] Querques G, Leveziel N, Benhamou N, Voigt M, Soubrane G, Souied EH (2006). Analysis of retinal flecks in fundus flavimaculatus using optical coherence tomography. Br J Ophthalmol.

[38] Stöhr H, Marquardt A, White K, Weber BH (2000). cDNA cloning and genomic structure of a novel gene (C11orf9) localized to chromosome 11q12--&gt;q13.1 which encodes a highly conserved, potential membrane-associated protein. Cytogenet Cell Genet.

[39] O'Gorman S, Flaherty WA, Fishman GA, Berson EL (1988). Histopathologic findings in Best's vitelliform macular dystrophy. Arch Ophthalmol.

[40] Weingeist TA, Kobrin JL, Watzke RC (1982). Histopathology of Best's macular dystrophy. Arch Ophthalmol.

[41] Petrukhin K, Koisti MJ, Bakall B, Li W, Xie G, Marknell T (1998). Identification of the gene responsible for Best macular dystrophy. Nat Genet.

[42] Do P, Ferrucci S (2006). Adult-onset foveomacular vitelliform dystrophy. Optometry.

[43] (2021). Adult-onset vitelliform macular dystrophy | Genetic and Rare Diseases Information Center (GARD) - an NCATS Program. https://rarediseases.info.nih.gov/diseases/10909/adult-onset-vitelliform-macular-dystrophy.

[44] Shah D, Saurabh K, Roy R (2018). Multimodal imaging in multifocal Best disease. Indian J Ophthalmol.

[45] Cusumano A, Falsini B, Giardina E, Cascella R, Sebastiani J, Marshall J (2019). Doyne honeycomb retinal dystrophy - functional improvement following subthreshold nanopulse laser treatment: a case report. J Med Case Rep.

[46] Fu L, Garland D, Yang Z (2007). The R345W mutation in EFEMP1 is pathogenic and causes AMD-like deposits in mice. Hum Mol Genet.

[47] Silvestri G, Sillery E, Henderson DC, Brogan PJ, Silvestri V (2005). Prevalence of drusen and drusen size in young adults. Invest Ophthalmol Vis Sci.

[48] Silvestri G, Williams MA, McAuley C (2012). Drusen prevalence and pigmentary changes in Caucasians aged 18-54 years. Eye (Lond).

[49] (2021). Drusen diagnosis and treatment - American Academy of Ophthalmology. https://www.aao.org/eye-health/diseases/drusen-treatment.

[50] Gregory CY, Evans K, Wijesuriya SD, Kermani S, Jay MR, Plant C (1996). The gene responsible for autosomal dominant Doyne’s honeycomb retinal dystrophy (DHRD) maps to chromosome 2p16. Hum Mol Genet.

[51] Manes G, Joly W, Guignard T (2017). A novel duplication of PRMD13 causes North Carolina macular dystrophy: overexpression of PRDM13 orthologue in drosophila eye reproduces the human phenotype. Hum Mol Genet.

[52] Hermsen VM, Judisch GF (1984). Central areolar pigment epithelial dystrophy. Ophthalmologica.

[53] Khurana RN, Sun X, Pearson E, Yang Z, Harmon J, Goldberg MF, Zhang K (2009). A reappraisal of the clinical spectrum of North Carolina macular dystrophy. Ophthalmology.

[54] Cipriani V, Silva RS, Arno G (2017). Duplication events downstream of IRX1 cause North Carolina macular dystrophy at the MCDR3 locus. Sci Rep.

[55] Small KW (1989). North Carolina macular dystrophy, revisited. Ophthalmology.

[56] Small KW, Vincent AL, Knapper CL, Shaya FS (2019). Congenital toxoplasmosis as one phenocopy of North Carolina Macular Dystrophy (NCMD/MCDR1). Am J Ophthalmol Case Rep.

[57] Small KW, Tran EM, Small L, Rao RC, Shaya F (2019). Multimodal imaging and functional testing in a North Carolina macular disease family: toxoplasmosis, fovea plana, and torpedo maculopathy are phenocopies. Ophthalmol Retina.

[58] Silva RS, Arno G, Cipriani V (2019). Unique noncoding variants upstream of PRDM13 are associated with a spectrum of developmental retinal dystrophies including progressive bifocal chorioretinal atrophy. Hum Mutat.

[59] Small KW (1998). North Carolina macular dystrophy: clinical features, genealogy, and genetic linkage analysis. Trans Am Ophthalmol Soc.

[60] Prensky JG, Bresnick GH (1983). Butterfly-shaped macular dystrophy in four generations. Arch Ophthalmol.

[61] Namburi P, Khateb S, Meyer S (2020). A unique PRDM13-associated variant in a Georgian Jewish family with probable North Carolina macular dystrophy and the possible contribution of a unique CFH variant. Mol Vis.

[62] Agarwal A, Patel P, Adkins T, Gass JD (2005). Spectrum of pattern dystrophy in pseudoxanthoma elasticum. Arch Ophthalmol.

[63] Agarwal A (2011). Gass’ atlas of macular diseases. https://books.google.co.uk/books?id=8uRKhLIIhxsC.

[64] Zhang K, Garibaldi DC, Li Y, Richard Green W, Zack DJ (2002). Butterfly-shaped pattern dystrophy: a genetic, clinical, and histopathological report. Arch Ophthalmol.

[65] Saksens NT, van Huet RA, van Lith-Verhoeven JJ, den Hollander AI, Hoyng CB, Boon CJ (2015). Dominant cystoid macular dystrophy. Ophthalmology.

[66] (2021). Diabetic maculopathy & diabetic macular oedema. https://www.diabetes.co.uk/diabetes-complications/diabetic-maculopathy.html.

[67] Cheung N, Mitchell P, Wong TY (2010). Diabetic retinopathy. Lancet.

[68] Flaxel CJ, Adelman RA, Bailey ST, Fawzi A, Lim JI, Vemulakonda GA (2020). Diabetic Retinopathy Preferred Practice Pattern®. Ophthalmology [Internet.

[69] Browning DJ, Stewart MW, Lee C (2018). Diabetic macular edema: evidence-based management. Indian J Ophthalmol.

[70] Tudor SM, Hamman RF, Baron A, Johnson DW, Shetterly SM (1998). Incidence and progression of diabetic retinopathy in Hispanics and non-Hispanic whites with type 2 diabetes. San Luis Valley Diabetes Study, Colorado. Diabetes Care.

[71] Klein R, Klein BE, Moss SE, Cruickshanks KJ (1995). The Wisconsin epidemiologic study of diabetic retinopathy. XV. The long-term incidence of macular edema. Ophthalmology.

[72] Saxena S, Akduman L, Meyer CH (2021). External limiting membrane: retinal structural barrier in diabetic macular edema. Int J Retina Vitreous.

[73] Melles RB, Marmor MF (2014). The risk of toxic retinopathy in patients on long-term hydroxychloroquine therapy. JAMA Ophthalmol.

[74] Marshall E, Robertson M, Kam S, Penwarden A, Riga P, Davies N (2021). Prevalence of hydroxychloroquine retinopathy using 2018 Royal College of Ophthalmologists diagnostic criteria. Eye (Lond).

[75] Bae EJ, Kim KR, Tsang SH, Park SP, Chang S (2014). Retinal damage in chloroquine maculopathy, revealed by high resolution imaging: a case report utilizing adaptive optics scanning laser ophthalmoscopy. Korean J Ophthalmol.

[76] Song W, Muste JC, Greenlee TE, Singh RP, Song W, Muste JC (2020). Chloroquine and hydroxychloroquine toxicity. Am J Ophthal Clinical Trials.

[77] Khan MJ, Papakostas T, Kovacs K, Gupta MP (2020). Drug-induced maculopathy. Curr Opin Ophthalmol.

[78] (2022). Crystalline retinopathy - EyeWiki. https://eyewiki.aao.org/Crystalline_Retinopathy.

[79] Moorthy RS, Lyon AT, Rabb MF, Spaide RF, Yannuzzi LA, Jampol LM (1998). Idiopathic polypoidal choroidal vasculopathy of the macula. Ophthalmology.

[80] Shechtman D, Tyler JA (2004). Idiopathic polypoidal choroidal vasculopathy (IPCV) presenting with simultaneous choroidal neovascular membrane (CNM). Optom Vis Sci.

[81] Ye H, Zhang Q, Liu X (2014). Prevalence of age-related macular degeneration in an elderly urban Chinese population in China: the Jiangning Eye Study. Invest Ophthalmol Vis Sci.

[82] Wong CW, Yanagi Y, Lee WK, Ogura Y, Yeo I, Wong TY, Cheung CM (2016). Age-related macular degeneration and polypoidal choroidal vasculopathy in Asians. Prog Retin Eye Res.

[83] Kondo N, Honda S, Ishibashi K, Tsukahara Y, Negi A (2007). LOC387715/HTRA1 variants in polypoidal choroidal vasculopathy and age-related macular degeneration in a Japanese population. Am J Ophthalmol.

[84] Liang XY, Chen LJ, Ng TK (2014). FPR1 interacts with CFH, HTRA1 and smoking in exudative age-related macular degeneration and polypoidal choroidal vasculopathy. Eye (Lond).

[85] Takahashi K, Jiang XC, Sakai N (1993). A missense mutation in the cholesteryl ester transfer protein gene with possible dominant effects on plasma high density lipoproteins. J Clin Invest.

[86] Kalogeropoulos D, Ch'ng SW, Lee R (2019). Optic disc pit maculopathy: a review. Asia Pac J Ophthalmol (Phila).

[87] Al-Mohtaseb Z, Foroozan R (2012). Congenital optic disc anomalies. Int Ophthalmol Clin.

[88] Wang Y, Xu L, Jonas JB (2006). Prevalence of congenital optic disc pits in adult Chinese: the Beijing Eye Study. Eur J Ophthalmol.

[89] Rossi S, De Rosa G, D'Alterio FM, Orrico A, Banfi S, Testa F, Simonelli F (2017). Intrafamilial heterogeneity of congenital optic disc pit maculopathy. Ophthalmic Genet.

[90] Jain N, Johnson MW (2014). Pathogenesis and treatment of maculopathy associated with cavitary optic disc anomalies. Am J Ophthalmol.

[91] Kranenburg EW (1960). Crater-like holes in the optic disc and central serous retinopathy. Arch Ophthalmol.

[92] Brockhurst RJ (1975). Optic pits and posterior retinal detachment. Trans Am Ophthalmol Soc.

[93] Shah SD, Yee KK, Fortun JA, Albini T (2014). Optic disc pit maculopathy: a review and update on imaging and treatment. Int Ophthalmol Clin.

[94] Healey PR, Mitchell P (2008). The prevalence of optic disc pits and their relationship to glaucoma. J Glaucoma.

[95] Abdellah MM, Mostafa EM, Anber MA, El Saman IS, Eldawla ME (2019). Solar maculopathy: prognosis over one year follow up. BMC Ophthalmol.

[96] Hossein M, Bonyadi J, Soheilian R, Soheilian M, Peyman GA (2011). Spectral-domain optical coherence tomography features of mild and severe acute solar retinopathy. Ophthalmic Surg Lasers Imaging.

[97] Jain A, Desai RU, Charalel RA, Quiram P, Yannuzzi L, Sarraf D (2009). Solar retinopathy: comparison of optical coherence tomography (OCT) and fluorescein angiography (FA). Retina.

[98] Gregory-Roberts E, Chen Y, Harper CA, Ong T, Maclean MA, Fagan XJ (201512021). Solar retinopathy in children. J Am Assoc Pediatr Ophthalmol Strabismus.

[99] Simakurthy S, Tripathy K (2023). Valsalva retinopathy. StatPearls [Internet].

[100] Mukherjee C, Kumar A, Mitra A (2018). Valsalva maculopathy: to treat or not to treat. Oman J Ophthalmol.

[101] Chapman-Davies A, Lazarevic A (2002). Valsalva maculopathy. Clin Exp Optom.

[102] Sakamoto SI, Makino S, Tampo H (2014). Double ring sign at the macula in a patient with Valsalva retinopathy. QJM.

[103] García Fernández M, Navarro JC, Castão CG (2012). Long-term evolution of Valsalva retinopathy: a case series. J Med Case Rep.

[104] Hanazono G, Shinoda K, Obazawa M, Imamura Y, Matsumoto SC, Satofuka S (2013). Valsalva retinopathy developing during Japanese cheerleading training ('ouendan’). Retin Cases Brief Rep.

[105] Bar-Sela SM, Moisseiev J (2007). Valsalva retinopathy associated with vigorous dancing in a discotheque. Ophthalmic Surg Lasers Imaging.

[106] Sheikh SA, Untoo RA, Lone IA, Shaheen N (2010). Maculopathy: a rare association of the Valsalva manoeuvre (Valsalva maculopathy). BMJ Case Rep.

[107] Brody S, Erb C, Veit R, Rau H (1999). Intraocular pressure changes: the influence of psychological stress and the Valsalva maneuver. Biol Psychol.

[108] Cheloni R, Gandolfi SA, Signorelli C, Odone A (2019). Global prevalence of diabetic retinopathy: protocol for a systematic review and meta-analysis. BMJ Open.

[109] Ramshekar A, Wang H, Hartnett ME (2021). Regulation of Rac1 activation in choroidal endothelial cells: Insights into mechanisms in age-related macular degeneration. Cells.

[110] (2018). Age-related macular degeneration: diagnosis and management. https://pubmed.ncbi.nlm.nih.gov/29400919/.

[111] Marques AP, Ramke J, Cairns J (2021). Global economic productivity losses from vision impairment and blindness. EClinicalMedicine.

[112] Burton MJ, Ramke J, Marques AP (2021). The Lancet Global Health Commission on Global Eye Health: vision beyond 2020. Lancet Glob Health.

[113] Liew G, Michaelides M, Bunce C (2014). A comparison of the causes of blindness certifications in England and Wales in working age adults (16-64 years), 1999-2000 with 2009-2010. BMJ Open.

[114] Thomas RL, Luzio SD, North RV, Banerjee S, Zekite A, Bunce C, Owens DR (2017). Retrospective analysis of newly recorded certifications of visual impairment due to diabetic retinopathy in Wales during 2007-2015. BMJ Open.

[115] (2022). IDF Diabetes Atlas. https://diabetesatlas.org/.

[116] (2024). Facts and figures | European Blind Union. https://www.euroblind.org/about-blindness-and-partial-sight/facts-and-figures.

[117] (2018). Economic costs of diabetes in the U.S. in 2017. Diabetes Care.

[118] Patel S (2018). Medicare spending on anti-vascular endothelial growth factor medications. Ophthalmol Retina.

[119] Swenor BK, Lee MJ, Varadaraj V, Whitson HE, Ramulu PY (2020). Aging with vision loss: a framework for assessing the impact of visual impairment on older adults. Gerontologist.

[120] van der Aa HP, Comijs HC, Penninx BW, van Rens GH, van Nispen RM (2015). Major depressive and anxiety disorders in visually impaired older adults. Invest Ophthalmol Vis Sci.

[121] Chiang PPC, Zheng Y, Wong TY, Lamoureux EL (2013). Vision impairment and major causes of vision loss impacts on vision-specific functioning independent of socioeconomic factors. Ophthalmology.

[122] The impact of pediatric vision disorders in adulthood: Queen Mary University Journal Titles. https://web.s.ebscohost.com/pfi/detail/detail.

[123] (2015). Transforming our world: the 2030 agenda for sustainable development. https://sustainabledevelopment.un.org/post2015/transformingourworld/publication.

[124] Lu LJ, Liu J, Adelman RA (2017). Novel therapeutics for Stargardt disease. Graefes Arch Clin Exp Ophthalmol.

[125] Parker MA, Choi D, Erker LR (2016). Test-retest variability of functional and structural parameters in patients with Stargardt disease participating in the SAR422459 gene therapy trial. Transl Vis Sci Technol.

[126] Schwartz SD, Regillo CD, Lam BL, Eliott D, Rosenfeld PJ, Gregori NZ (2015). Human embryonic stem cell-derived retinal pigment epithelium in patients with age-related macular degeneration and Stargardt’s macular dystrophy: follow-up of two open-label phase 1/2 studies. Lancet.

[127] Khan KN, Mahroo OA, Islam F, Webster AR, Moore AT, Michaelides M (2017). Functional and anatomical outcomes of choroidal neovascularization complicating Best1-related retinopathy. Retina.

[128] Leasher JL, Bourne RR, Flaxman SR (2016). Global estimates on the number of people blind or visually impaired by diabetic retinopathy: a meta-analysis from 1990 to 2010. Diabetes Care.

[129] Wong TY, Sun J, Kawasaki R (2018). Guidelines on diabetic eye care: the International Council of Ophthalmology recommendations for screening, follow-up, referral, and treatment based on resource settings. Ophthalmology.

[130] Tan GS, Cheung N, Simó R, Cheung GC, Wong TY (2017). Diabetic macular oedema. Lancet Diabetes Endocrinol.

[131] Chan WC, Wu AC, Tsai S, Chen LJ (2022). Current treatments of diabetic macular edema. Int J Gerontol.

[132] Kim EJ, Lin WV, Rodriguez SM, Chen A, Loya A, Weng CY (2019). Treatment of diabetic macular edema. Curr Diab Rep.

[133] Flaxel CJ, Adelman RA, Bailey ST, Fawzi A, Lim JI, Vemulakonda GA, Ying GS (2020). Diabetic Retinopathy Preferred Practice Pattern®. Ophthalmology.

[134] (2013). Lutein + zeaxanthin and omega-3 fatty acids for age-related macular degeneration: the Age-Related Eye Disease Study 2 (AREDS2) randomized clinical trial. JAMA.

[135] Stahl A (2020). The diagnosis and treatment of age-related macular degeneration. Dtsch Arztebl Int.

[136] (2023). Managing age-related macular degeneration - NICE Pathways. https://pathways.nice.org.uk/pathways/age-related-macular-degeneration.

[137] Upasani D, Dhingra N (2021). Ten-year outcome of anti-vascular endothelial growth factor treatment for neovascular age-related macular degeneration. Indian J Ophthalmol.

[138] Wecker T, Grundel B, Reichl S (2019). Anti-VEGF injection frequency correlates with visual acuity outcomes in pro re nata neovascular AMD treatment. Sci Rep.

[139] Campa C (2020). New anti-VEGF drugs in ophthalmology. Curr Drug Targets.

[140] Gahn GM, Khanani AM (2018). New therapies of neovascular AMD beyond anti-VEGF injections. Vision (Basel).

[141] Wecker T, Ehlken C, Bühler A, Lange C, Agostini H, Böhringer D (2017). Five-year visual acuity outcomes and injection patterns in patients with pro-re-nata treatments for AMD, DME, RVO and myopic CNV. Br J Ophthalmol.

[142] (2024). Quality statement 3: treatment - late age-related macular degeneration (wet active) | serious eye disorders | Quality standards | NICE. https://www.nice.org.uk/guidance/qs180/chapter/quality-statement-3-treatment-late-age-related-macular-degeneration-wet-active.

